# Chemical structures, biological activities, and biosynthetic analysis of secondary metabolites of the Diatrypaceae family: A comprehensive review

**DOI:** 10.1080/21501203.2024.2341648

**Published:** 2024-04-24

**Authors:** Shi-Jie Kang, Ling Zhao, Haiqiang Wang, Jin-Ming Gao, Jianzhao Qi

**Affiliations:** a Shaanxi Key Laboratory of Natural Products & Chemical Biology, College of Chemistry & Pharmacy, Northwest A&F University, Yangling, China; b Department of Pharmacy, School of Medicine, Xi’an International University, Xi’an, China

**Keywords:** Diatrypaceae, pimarane diterpenoids, cytosporins, biosynthetic analysis

## Abstract

The family Diatrypaceae is a less well-known group within the order Xylariales (Ascomycota). Initially, the focus on its metabolites was related to the pathogenicity of one of its members, *Eutypa lata*. To date, a total of 254 natural products have been identified from Diatrypaceae strains. These compounds include terpenoids, sterols, polyketones, phenols, and acetylene aromatic compounds, which have shown anticancer, cytotoxic, anti-inflammatory, antimicrobial, and antiviral activities. The complex and diverse structural types, along with the diverse bioactivities, highlight the potential of Diatrypaceae as a valuable source of bioactive natural products. In this review, a deep analysis of the biosynthesis of pimarane diterpenes and scoparasin-type cytochalasins is provided, coupled with a compilation of the biosynthetic pathways of aromatic acetylene compounds in filamentous fungi. This comprehensive review not only enhances our understanding of the natural product chemistry, biological activities, and biosynthesis of secondary metabolites from the Diatrypaceae family but also promotes the exploitation and development of important bioactive compounds and potential strains.

## 1. Introduction

The Diatrypaceae is a family of fungi in the order Xylariales (Ascomycota). The species within the Diatrypaceae family were relatively unknown until recently. Initially, Diatrypaceae was thought to be the only family in the order Diatrypales, consisting of only nine genera (Kirk et al. 2001). However, in 2020, Diatrypaceae was extensively revised and more than 20 new genera were included (Long et al. 2021). Unlike other ascomycete taxa, there are no clear morphological characteristics that can be used to distinguish members of the Diatrypaceae at the genus or species level. Currently, differentiation is commonly achieved through the use of ITS sequences derived from rDNA and other molecular genetic characters (Acero et al. 2004).

While our understanding of the taxonomy and species diversity within the Diatrypaceae family is limited, there has been extensive research into their pathogenicity and agricultural harm. Diatrypaceae species are widely distributed in terrestrial and marine environments worldwide, with some species causing significant agricultural damage as plant pathogens. For example, Diatrypaceae has long been recognised as the causal agent of grapevine dieback disease worldwide and has also been reported as a pathogen of fruit trees and woody plants in Europe, the USA, and Africa (Moyo et al. 2018; Long et al. 2021). *Eutypa* and *Eutypella*, common plant pathogens, were initially identified as grapevine pathogens but have recently been found to be pathogenic on a wide range of hosts including apricots and plums (Moyo et al. 2018). *Cryptosphaeria*, another genus within the Diatrypaceae family, causes dieback and discoloration of poplar and is a significant threat to poplar growth in China, Europe, and the United States (Zhao et al. 2006). *Eutypa* dieback disease, caused by *E. lata*, affects the normal growth of grapes and various other woody fruit plants, perpetuating a perennial canker disease (Jiménez-Teja et al. 2006).

The natural product chemistry of Diatrypaceae metabolites has evolved in the course of research into the agricultural pathogenesis of Diatrypaceae. Aromatic acetylenic compounds, reported as early as 1989, are thought to be toxic molecules produced by *E. lata*, the causal agent of grape “dying-arm” disease (Renaud et al. 1989a, 1989b). Subsequent studies have shown that *E. lata* has an abundant production capacity for secondary metabolites that contribute to the damage that *E. lata* causes to plants as a pathogen (Jiménez-Teja et al. 2006). *Eutypella* is another group in the family Diatrypaceae that is capable of producing secondary metabolites with various structures (Zhou et al. 2022), either of marine origin (Sun et al. 2012b; Liu et al. 2017) or as a plant endophyte (Zhu et al. 2021).

Although the presence of a wide range of secondary metabolites in members of the Diatrypaceae family has been established, our overall understanding of these compounds remains limited. This paper aims to provide the first systematic summary of the secondary metabolites found in this family and their corresponding biological activities. Through a comprehensive review, a total of 254 compounds belonging to the Diatrypaceae family were compiled. These compounds were classified into six different groups based on their structural features and biosynthetic patterns, including terpenoids, cytochalasins, and others. Notably, terpenoids make up more than half of the total, with 140 compounds falling into this category. Significantly, many of these metabolites exhibit remarkable biological activities, such as anti-cancer, cytotoxic, antimicrobial, anti-inflammatory, and antiviral properties, suggesting their potential as promising candidates for future clinical drug development. In addition, this review provides an in-depth analysis of the enzymes associated with the biosynthesis of scoparasins and pimarane diterpenes, as well as a summary of advances in the understanding of the biosynthesis of aromatic alkyne compounds. In conclusion, this comprehensive review is of immense value in further exploring the natural product chemistry, pharmacological activity, and biosynthesis of secondary metabolites in the Diatrypaceae family, paving the way for future scientific endeavours.

## 2. Chemical structures

Out of 254 compounds, more than half of these metabolites belong to terpenoids (including four monoterpenes, 82 sesquiterpenes, 43 diterpenes, two triterpenes, and nine meroterpenes) and steroids (seven ergosterols), while the remaining compounds encompass diverse categories such as cytochalasins (ten compounds), polyketones and phenolics (45 compounds), diketopiperazines (26 compounds), aromatic acetylenic compounds (16 compounds), as well as several miscellaneous compounds. The names, producers, isolated sources, and biological activities of these compounds are given in Table S1.

### 2.1. Terpenoids and steroids

**Figure 1.**
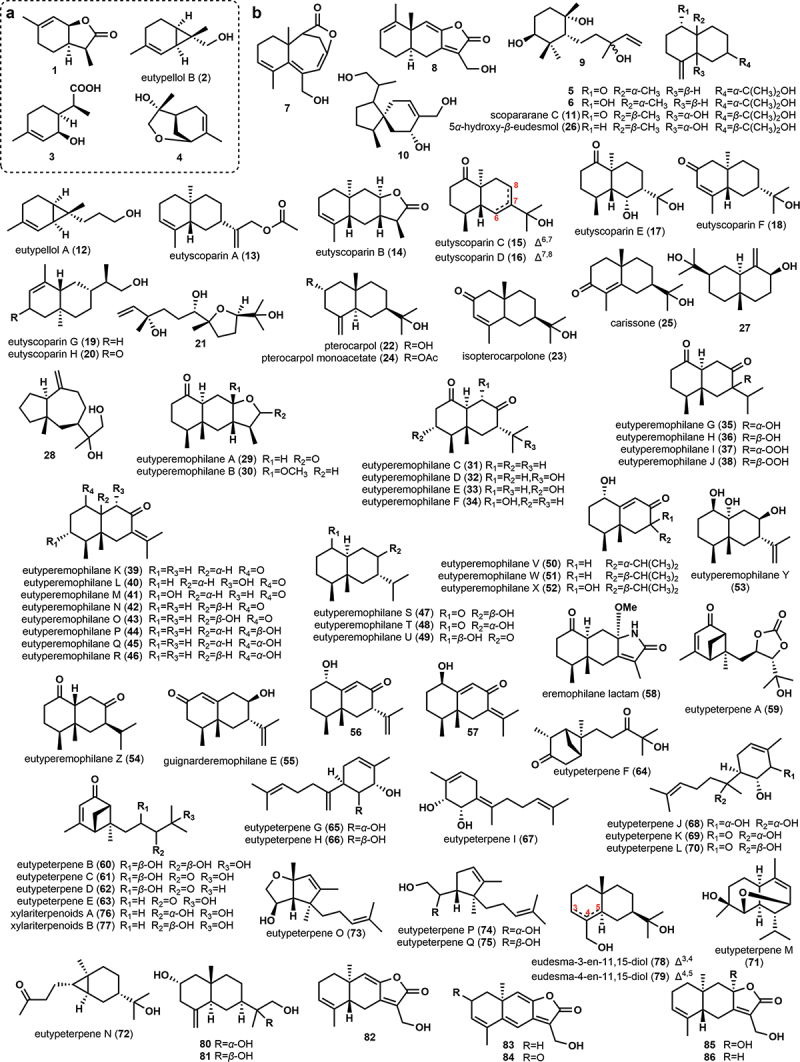
Chemical structures of monoterpenes (a) and sesquiterpenes (b) from the family Diatrypaceae.

A total of four monoterpene compounds (Figure 1a) have been identified in the metabolites of the Diatrypaceae family. Among them, (3*S*,3a*R*,7a*S*)-3a,4,5,7a-tetrahydro-3,6-dimethylbenzofuran-2(3*H*)-one (**1**), a dimethylbenzofuran lactone derived from a menthane-type monoterpene, was isolated from the fermentation broth of the marine fungus *Eutypella scoparia* FS26 (Sun et al. 2011b, 2012b). Another rare compound, eutypellol B (**2**), which is a 7-methyl oxidised derivative of 2-carene, was also found in the culture broth of the marine sediment-derived fungus *Eutypella scoparia* FS46. In addition, a previously undescribed monoterpene, 2-(2-hydroxy-4-methylcyclohex-3-enyl) propanoic acid (**3**), and a known compound, 2,9-epoxy-p-menth-6-en-9-ol (**4**), were also identified in the same culture broth (Liu et al. 2017).

A total of 82 sesquiterpenes (Figure 1b) have been isolated from the family Diatrypaceae, which can be categorised into different structural types including eudesmane, monocyclofarnesane, acorane, nerolidol, quadrijugol, eremophilane, bergamotane, bisabolane, cadinene, carabrane, and other types. Two *ent*-eudesmane sesquiterpenes, *ent*-4(15)-eudesmen-11-ol-1-one (**5**) and *ent*-4(15)-eudesmen-1*α*,11-diol (**6**), were isolated from the fermentation broth of the endophytic fungus *Eutypella* sp. BCC 13199 (Isaka et al. 2009). The sesquiterpene *eut*-Guaiane (**7**) was discovered as a new sesquiterpene lactone from the culture broth of *Eutypella* sp. D-1 (Lu et al. 2015). Subsequently, a sesquiterpene also named *eut*-Guaiane (**8**) was isolated and characterised from the same culture broth, but its structure was different from the former (Zhou et al. 2017). Novel monocyclofarnesane-type sesquiterpene 3,7,10-trihydroxy-6,11-cyclofarnes-1-ene (**9**), acorane-type sesquiterpene 8-(hydroxymethyl)-1-(1-hydroxy-1-methylethyl)-4-methylspiro[4.5]dec-8-en-7-ol (**10**), *β*-eudesmol type sesquiterpene scopararane C (**11**), norsesquiterpenoid of sequicarene family eutypellol A (**12**), and eudesmane-type sesquiterpenes eutyscoparins A– H (**13–20**) as well as known sesquiterpenes including one nerolidol-type sesquiterpene *rel*-(3*S*,6*S*,7*R*,10*R*)-7,10-epoxy-3,7,11-trimethyldodec-1-ene-3,6,11-triol (**21**), seven eudesmane-type sesquiterpenes *ent*-4(15)-eudesmen-11-ol-1-one (**5**), pterocarpol (**22**), isopterocarpolone (**23**), pterocarpol monoacetate (**24**), carissone (**25**), 5*α*-hydroxy-*β*-eudesmol (**26**), (3*β*)-eudesm-4(14)-ene-3,11-diol (**27**), a quadrijugol-type sesquiterpene ambrosanoli-10(14)-en-11,12-diol (**28**), and **8**, were isolated and identified from the metabolites of the genus *Eutypella* (Sun et al. 2011b, 2012b; Qi et al. 2015; Liu et al. 2017; Zhang et al. 2021a), and these producers include *E. scoparia* FS26, *E. scoparia* FS46., *Eutypella scoparia* SCBG-8, and *E. scoparia* 1–15 (Sun et al. 2011b, 2012b; Qi et al. 2015; Liu et al. 2017; Zhang et al. 2021a).

A total of 26 novel eremophilane-type sesquiterpenes, named eutyperemophilanes A–Z (**29**–**54**), were discovered from the modified rice medium of the fungus *Eutypella* sp. MCCC 3A00281 by chemical epigenetic manipulation. In addition, four previously reported terpenoids, guignarderemophilane E (**55**), 1*α*-hydroxyeremophila-9,11-dien-8-one (**56**), 1*β*-hydroxyeremophila-7(11),9-dien-8-one (**57**), and eremophilane lactam (**58**), were also identified. Interestingly, the production of these metabolites was found to be dependent on the presence of a histone deacetylase inhibitor, suberohydroxamic acid (SBHA), as the absence of SBHA in the culture medium resulted in the complete absence of these metabolites (Niu et al. 2018). Further studies on the fungus *Eutypella* sp. MCCC 3A00281 showed that simultaneous treatment with SBHA and 5-azacytidine, led to the isolation and identification of seventeen sesquiterpenes, known as eutypeterpenes A–Q (**59–75**), together with four described sesquiterpenes, xylariterpenoids A–B (**76–77**), eudesma-3-en-11,15-diol (**78**), and eudesma-4-en-11,15-diol (**79**) (Niu et al. 2021). A pair of known epimers, (11*R*)-2,11,12-trihydroxy-*β*-selinene (**80**) and (11*S*)-2,11,13-trihydroxy-*β*-selinene (**81**), was obtained from the rice medium of *Eutypella* sp. ZZ2 (Liao et al. 2017). In addition, four new 12,8-eudesmanolides, namely 13-hydroxy-3,8,7(11)-eudesmatrien-12,8-olide (**82**), 13-hydroxy-3,5,8,7(11)-eudesmatetraen-12,8-olide (**83**), 2-one-13-hydroxy-3,5,8,7(11)-eudesmatetraen-12,8-olide (**84**), and 8,13-dihydroxy-3,7(11)-eudesmadien-12,8-olide (**85**), were isolated and purified from the rice medium of *Eutypella* sp. 1–15, along with the known 13-hydroxy-3,7(11)-eudesmadien-12,8-olide (**86**) (Wang et al. 2017).

**Figure 2.**
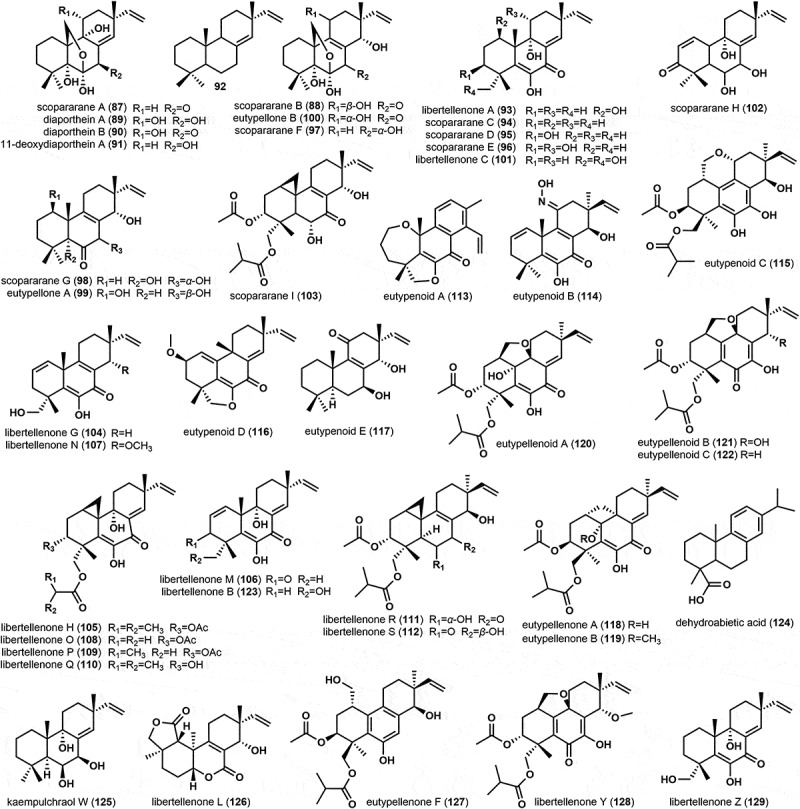
Chemical structures of diterpenes from the family Diatrypaceae.

A total of 43 diterpenoids (Figure 2), all of the pimarane diterpenes, have been isolated from the Diatrypaceae family. Scopararanes A and B (**87**–**88**) and diaportheins A and B (**89**–**90**) were isolated from the fermentation broth of *Eutypella scoparia* PSU-D44 (Pongcharoen et al. 2006). In addition, a new structure named 11-deoxydiaporthein A (**91**) was discovered alongside compounds **87**, **89**–**90** from the filtered cultivation medium of *Cryptosphaeria eunomia* var. *eunomia* (Yoshida et al. 2007). Subsequently, isopimara-8(14),15-diene (**92**), libertellenone A (**93**), and **88**–**91** were identified from the culture broth and mycelium of *Eutypella scoparia* (Schw.) Ell. et Ev (Sun et al. 2011a). Furthermore, **87**, **89**–**91** were also isolated from the culture broth of *Eutypella scoparia* PSU-H267 (Kongprapan et al. 2015). Five new oxygenised pimarane diterpenes, scopararanes C–G (**94**–**98**), were identified from the culture broth of *E. scoparia* FS26 (Sun et al. 2012a). It is worth noting that **91** is the first reported pimarane-type diterpene derived from the genus *C. eunomia* var. *eunomia*, while **91**–**93** are the first isolates from the genus *E. scoparia*. However, there exists a naming duplication between **11** and **94**.

Using a bioreactor, two novel diterpenes, eutypellones A and B (**99**–**100**), and three known diterpenes, **87**, **90**, and libertellenone C (**101**), were isolated from the culture broth of *Eutypella* sp. BCC 13199 (Isaka et al. 2009, 2011). Two novel diterpenes, scopararanes H and I (**102**–**103**), along with **89**–**91** and **93**–**94**, were isolated from the culture broth of *Eutypella* sp. FS46 (Liu et al. 2017). Nineteen new diterpenoids, libertellenones G and H (**104**–**105**), libertellenones M and N (**106**–**107**), libertellenones O–S (**108**–**112**), eutypenoids A–E (**113**–**117**), eutypellenones A and B (**118**–**119**), eutypellenoids A–C (**120**–**122**), together with the reported compounds **93**, **99**, **101**, libertellenone B (**123**), dehydroabietic acid (**124**), kaempulchraol W (**125**), and libertellenone L (**126**) were isolated from the fermentation broth of *Eutypella* sp. D-1 (Lu et al. 2014; Liu 2016; Zhang et al. 2016; Wang et al. 2018; Yu et al. 2018a, 2018b). In a subsequent study, using the one strain many compounds (OSMAC) strategy, which involved adding ethanol as a promoter in the culture broth, led to the isolation and identification of three new pimarane-type diterpenes, eutypellenone F (**127**), libertellenone Y (**128**), and libertellenone Z (**129**), and four known diterpenes, **93**, **99**, **111**, and **123** (Ning et al. 2023).

**Figure 3.**
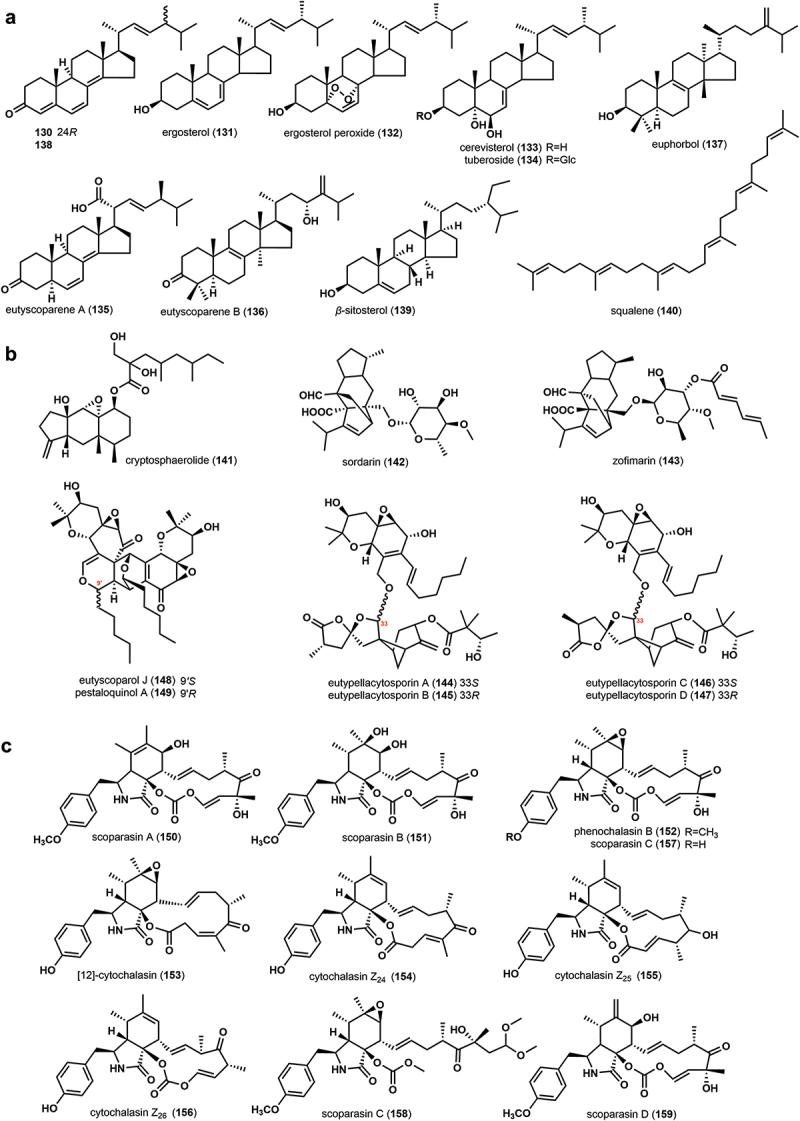
Chemical structures of triterpenes and steroids (a), meroterpenes (b), and cytochalasins (c) from the family Diatrypaceae.

Six ergosterol compounds, (22*E*,24*R*)-ergosta-4,6,8(14)-22-tetraen-3-one (**130**), ergosterol (**131**), ergosterol peroxide (**132**), cerevisterol (**133**), tuberoside (**134**) and eutyscoparene A (**135**), one new triterpenoid eutyscoparene B (**136**), one euphane triterpenoid euphorbol (**137**), two known steroids, (24)-epimeric mixtures of (22*E*)-ergosta-4,6,8(14)-22-tetraen-3-one (**138**) and *β*-sitosterol (**139**), and the acyclic triterpene precursor squalene (**140**) were isolated from the fermentation broth of the genus *Eutypella* (Sun et al. 2011a, 2011b, 2012b; Liu 2016; Zhang et al. 2021a), and these strain includes *E. scoparia* FS26, *E. scoparia* (Schw.) Ell. et Ev., *E, scoparia* SCBG-8, and *Eutypella* sp. D-1 (Sun et al. 2011a, 2011b, 2012b; Liu 2016; Zhang et al. 2021a). Ergosterol (**131**) has also been identified as a metabolite of *Eutypa* sp. (#424) (Lin et al. 2002). The structures of these compounds are shown in Figure 3(a).

Cryptosphaerolide (**141**), an ester-substituted eremophilane-type sesquiterpenoid, was identified from the saline fermentation of the marine-derived fungus *Cryptosphaeria* sp. CNL-523 (Oh et al. 2010). Two diterpenoid glycosides, sordarin (**142**) and zofimarin (**143**) were discovered and identified from *Diatrype stigma* and *Eutypa tetragona*, respectively (Vicente et al. 2009). It is noteworthy that this is the first report on the production of sordarin compounds by the family Diatrypaceae. Four novel meroterpenoids eutypellacytosporins A–D (**144**–**147**) were isolated from the solid rice medium of *Eutypella* sp. D-1. Noteworthy, these unusual compounds were probably produced by the induction of rice medium (Zhang et al. 2019). Later, a new meroterpenoid, eutyscoparol J (**148**), was isolated from the solid rice medium of endophytic *E. scoparia* SCBG-8, together with pestaloquinol A (**149**) (Zhang et al. 2021b). The structures of these meroterpenes are displayed in Figure 3(b).

### 2.2. Cytochalasins

Cytochalasins derive their name from the Greek words “kytos” and “chalasis”, meaning cell and relaxation, respectively. Cytochalasins are synthesised by a variety of fungal polyketide synthases-non-ribosomal peptide synthetases (PKS-NRPS) and have a wide range of unique biological functions. The genus *Eutypella* species is the only taxon that produces cytochalasins in the family Diatrypaceae, and these strains include *E. scoparia* PSU-D44, *E. scoparia* FS26, *Eutypella* sp. D-1, *E*. *scoparia* PSU-H267, *E. scoparia* 1–15, and *E. scoparia* SCBG-8 (Pongcharoen et al. 2006; Sun et al. 2011b, 2013; Liu et al. 2014; Kongprapan et al. 2015; Qi et al. 2015; Zhou et al. 2017; Zhang et al. 2021b). These cytochalasins (Figure 3(c)) include scoparasins A and B (**150**–**151**), phenochalasin B (**152**), [12]-cytochalasin (**153**), cytochalasins Z_24_–Z_26_ (**154**–**156**), scoparasin C (**157** and **158**) and scoparasin D (**159**) (Pongcharoen et al. 2006; Sun et al. 2011b, 2013; Liu et al. 2014; Kongprapan et al. 2015; Qi et al. 2015; Zhou et al. 2017; Zhang et al. 2021b). It is worth mentioning that **157** and **158** are two different structures reported by Kongprapan et al. (2015) and Qi et al. (2015), respectively, but share the nomenclature scoparasin C.

### 2.3. Polyketones and phenolics

**Figure 4.**
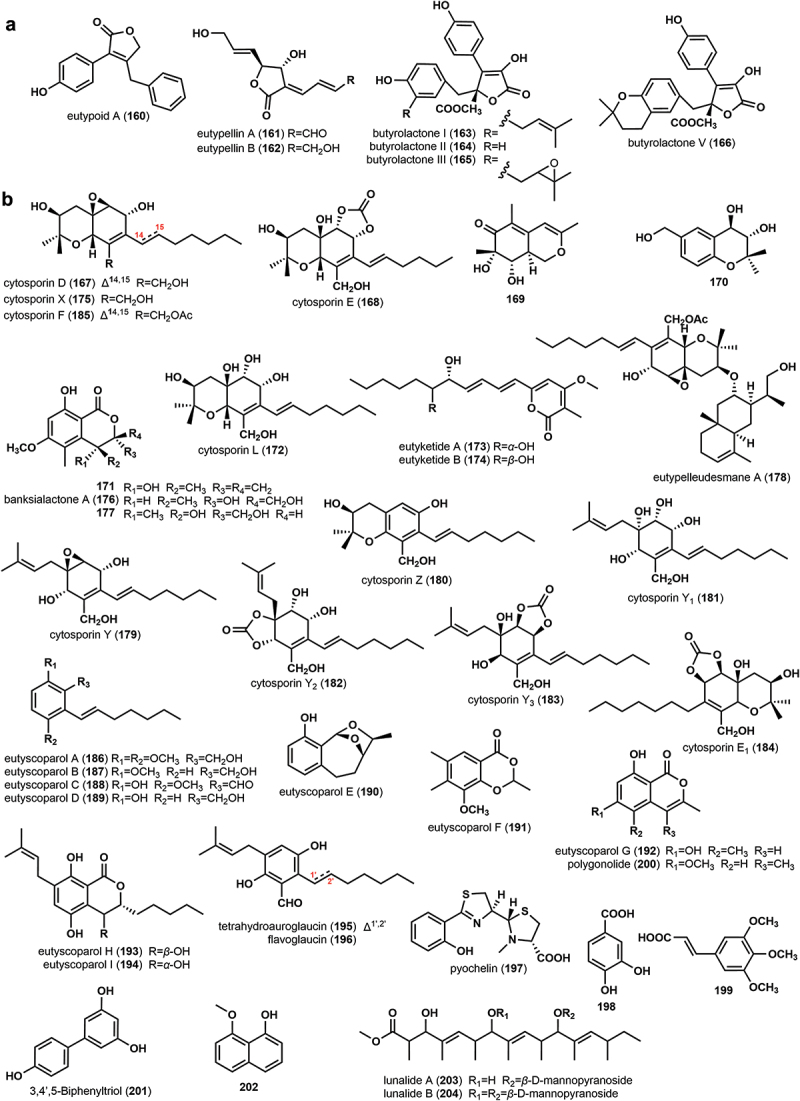
Chemical structures of *γ*-lactones (a) and other polyketones (b) from the family Diatrypaceae.

Seven *γ*-lactones (Figure 4(a)) were discovered in metabolites of the Diatrypaceae family. A novel *γ*-lactone, eutypoid A (**160**), was isolated from the culture broth of *Eutypa* sp. (#424) (Lin et al. 2002). Two new *γ*-lactones, eutypellins A (**161**) and B (**162**), were isolated from the fermentation broth of the endophytic fungus *Eutypella* sp. BCC 13199 (Isaka et al. 2009). Four reported compounds butyrolactones I–V (**163–166**) were obtained from the solid medium of *Eutypella* sp. ZZ2 (Liao et al. 2017).

Two cytosporin-related compounds, cytosporin D (**167**) and cytosporin E (**168**), were isolated from the culture broth of *Eutypella scoparia* ICB-OBX (Ciavatta et al. 2008). Two novel polyketides, 7,8-dihydroxy-3,5,7-trimethyl-8,8a-dihydro-1*H*-isochromen-6(7*H*)-one (**169**) and 6-(hydroxymethyl)-2,2-dimethyl-3,4-dihydro-2*H*-chromene-3,4-diol (**170**), were isolated from the fermentation broth of *E*. *scoparia* FS26 (Sun et al. 2013). The known compound (*R*)-3,4-dihydro-4,8-dihydroxy-6-methoxy-4,5-dimethyl-3-methyleneisochromen-1-one (**171**) was discovered and identified from the culture broth of *E*. *scoparia* PSU-D44 and *E. scoparia* PSU-H267 (Pongcharoen et al. 2006; Kongprapan et al. 2015). A new hexahydrobenzopyran derivative cytosporin L (**172**), together with **167**–**168** were isolated from the solid medium of *Eutypella* sp. ZZ2 (Liao et al. 2017). Compound **167** was also purified from the potato dextrose broth of *Eutypella* sp. D-1 (Zhang et al. 2019). Three new polyketides, eutyketides A and B (**173**–**174**) and cytosporin X (**175**), as well as four known compounds, **167**, **171**, banksialactone A (**176**), and 4,8-dihydroxy-3-(hydroxymethyl)-6-methoxy-4,5-dimethylisochroman-1-one (**177**), were obtained from the rice medium of *Eutypella scoparia* HBU-91 (Zhang et al. 2022a). Based on the OSMAC approach, seven new cytosporin derivatives, eutypelleudesmane A (**178**), cytosporin Y (**179**), cytosporin Z (**180**), cytosporin Y_1_ (**181**), cytosporin Y_2_ (**182**), cytosporin Y_3_ (**183**), and cytosporin E_1_ (**184**), together with five known biogenetically related analogs, **167**, **168**, **172**, **175**, and cytosporin F (**185**), were isolated from the rice solid medium and the defined solid medium of the Arctic-derived fungus *Eutypella* sp. D-1 (Yu et al. 2023).

Nine novel phenolic polyketide derivatives, eutyscoparols A–I (**186**–**194**), along with two related known compounds, tetrahydroauroglaucin (**195**) and flavoglaucin (**196**), were separated from the solid rice medium of the fungus *E. scoparia* SCBG-8 (Zhang et al. 2020, 2021b). Pyochelin (**197**), 3,4-dihydroxybenzoic acid (**198**), 3,4,5-trimethoxycinnamic acid (**199**), polygonolide (**200**) and 3,4’,5-Biphenyltriol (**201**), were discovered identified through Mass spectroscopy analysis as metabolites from the Iron-free culture broth of *E. lata* (Perez-Gonzalez et al. 2022). 8-methoxynaphthalen-1-ol (**202**) was isolated from the culture broth of *D. palmicola* MFLUCC 17-0313 (Tanapichatsakul et al. 2020). Two new glycosylated polyketides named lunalides A and B (**203**–**204**), were novel natural products produced by *Diatrype* sp. induced by the administration of a small molecule epigenetic modifier (5-azacytidine). Notably, the original uninduced strain was unable to produce these two new compounds (Williams et al. 2008). The structures of the above compounds are shown in Figure 4(b).

### 2.4. Diketopiperazines

**Figure 5.**
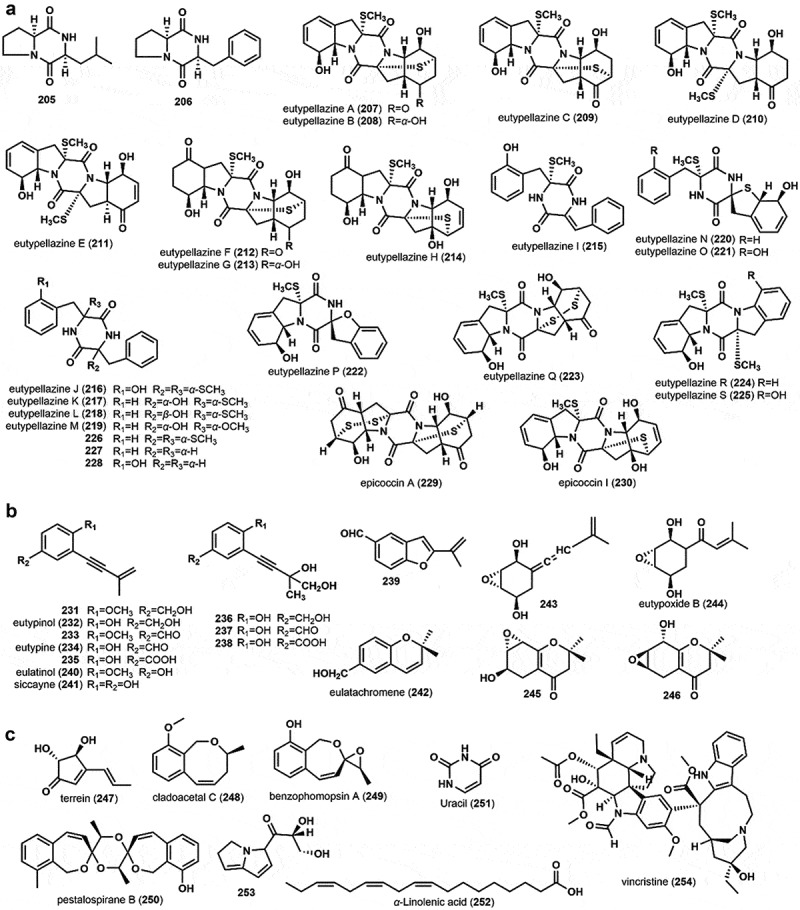
Chemical structures of diketopiperazines (a), aromatic acetylenic compounds (b), and miscellaneous compounds (c) from the family Diatrypaceae.

Two known cyclodipeptides, *cyclo*-(L-Pro-L-Leu) (**205**) and *cyclo*-(L-Pro-L-Phe) (**206**) were found in the culture broth of *E. scoparia* ICB-OBX (Ciavatta et al. 2008). In 2017, Niu et al. (2017a, 2017b) discovered and identified a series of thiodiketopiperazine alkaloids, eutypellazines A–S (**207**–**225**), in addition to several biosynthetic intermediates of these alkaloids, compounds **226**–**228**, and two known compounds, epicoccin A (**229**) and epicoccin I (**230**), from the rice medium of the deep-sea sediment derived fungus *Eutypella* sp. MCCC 3A00281. In particular, the spirocyclic analogues eutypellazines N–O (**220**–**221**), with a tetrahydrobenzothiophene moiety, were found for the first time from a wide-type fungus (Niu et al. 2017b). Except for **205** and **206**, they are all thiodiketopiperazine analogues. Their structures are displayed in Figure 5(a).

### 2.5. Aromatic acetylenic compounds

Eight new aromatic acetylenic metabolites, 4-Methoxy-3-(3-methylbut-3-en-1-ynyl)benzyl Alcohol (**231**), eutypinol (**232**), 4-Methoxy-3-(3-methylbut-3-en-1-ynyl)benzaldehyde (**233**), eutypine (**234**), eutypinic acid (**235**), 4-Hydroxy-3-(3,4-dihydroxy-3-methylbut-1-ynyl)benzyl Alcohol (**236**), 4-Hydroxy-3-(3,4-dihydroxy-3-methylbut-1-ynyl)benzaldehyde (**237**), and 4-Hydroxy-3-(3,4-dihydroxy-3-methylbut-1-ynyl)benzoic acid (**238**), as well as a benzofuran derivative, 5-formyl-2-(methylvinyl)[1]benzofuran (**239**), were isolated and identified from the culture medium of *E. lata* (Renaud et al. 1989b). Further research on the production of aromatic acetylenic metabolites by three different subspecies of *E. lata*, which include E120, E178, and E125, exhibited that metabolite composition and yields differed significantly between strains and with growth medium (Molyneux et al. 2002). Eulatinol (**240**) and siccayne (**241**) were obtained from the MYB medium of *E. lata* E120. Eutypine (**234**), eutypinol (**232**), and a chromene analog eulatachromene (**242**) of **232** were isolated from the PDB culture medium of *E. lata* E125. Meanwhile, none of these metabolites were observed in *E. lata* E178 (Molyneux et al. 2002). A novel allenic epoxycyclohexanes, 5-(3-methylbuta-1,3-dienylidene)-2,3-epoxycyclohexane-1,4-diol (**243**), in addition, a novel oxygenised cyclohexene oxide, eutypoxide B (**244**), along with two tetrahydrochromanone derivatives 6-hydroxy-2,2-dimethyl-5,6,7,8-tetrahydro-7,8-epoxychroman-4-one (**245**) and 8-hydroxy-2,2-dimethyl-5,6,7,8-tetrahydro-6,7-epoxychroman-4-one (**246**), have been isolated from the culture broth of *E. lata* (Pers: F.) TUL (Renaud et al. 1989a; Defrancq et al. 1992). The structures of these aromatic acetylenic compounds are shown in Figure 5(b).

### 2.6. Miscellaneous compounds

Terrein (**247**) was discovered and identified through mass spectroscopy analysis as metabolites from the Iron-free culture broth of *E. lata*, which was found to have iron-reducing abilities (Perez-Gonzalez et al. 2022). Three benzo[c]oxepin compounds, cladoacetal C (**248**), benzophomopsin A (**249**), and pestalospirane B (**250**), in which cladoacetal C (**248**) as a new natural product, were separated and purified from the rice medium of *Eutypella* sp. D-1 (Yu et al. 2020). Uracil (**251**) and *α*-Linolenic acid (**252**) were separated from the fermentation broth of *E. scoparia* FS26 and *Eutypella* sp. D-1, respectively (Sun et al. 2011b; Liu 2016). The crystal structure of a new substituted pyrrolizine alkaloid, (*R*)-1-(2,3-dihydro-1*H-*pyrrolizin-5-yl)-2,3-dihydroxypropan-1-one (**253**), was isolated from the broth of *Eutypella* sp. D-1 (Tan et al. 2017). Vincristine (**254**), a famous chemotherapy medication used to treat various types of cancer, has been isolated and identified from the fermentation broth of the endophytic fungus *Eutypella* spp. CrP14 in *Cathantanthus roseus*, with a yield of 53 ± 5.0 μg/L in the culture broth (Kuriakose et al. 2016). Interestingly, this is the first demonstrated fungal vincristine produced by *Eutypella* species. The structures of these compounds are shown in Figure 5(c).

## 3. Biological activities

**Figure 6.**
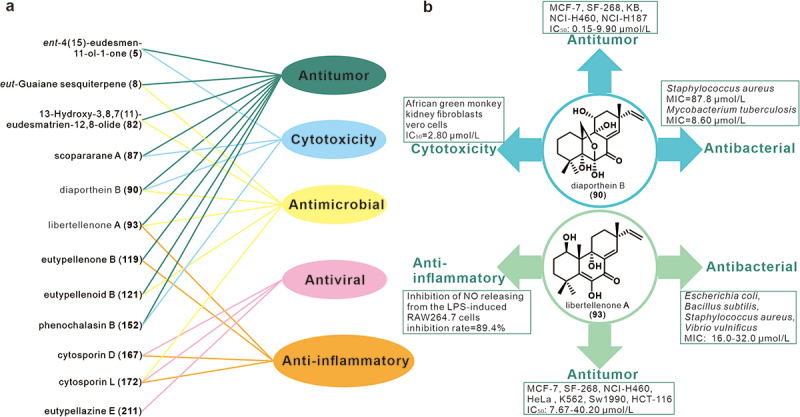
The biological activities of representative compounds (a), diaporthein B (**90**), and libertellenone A (**93**) (b) from the family Diatrypaceae.

Diatrypaceae family strains produce metabolites with diverse structures. Numerous studies have been reported on the biological activities and pharmacological mechanisms of Diatrypaceae secondary metabolites. The present review summaries these studies and finds that the bioactivities of Diatrypaceae secondary metabolites exhibit anticancer, antimicrobial, immunomodulatory, and antiviral activities (Figure 6(a)), with pimarane diterpenoids exhibiting a wide range of pharmacological properties (Figure 6(b)).

### 3.1. Anti-cancer, anti-tumour, and cytotoxic activities

A compilation and summary of the literature shows that a total of 43 compounds have inhibitory effects on a wide range of common cancer cells. The inhibitory activity of compounds **9**, **10**, **88**, **90**, **93**–**96**, **98**, **103**, **134**, **137**, **152**, and **153**, against three tumour cell lines, MCF-7, NCI-H460, and SF-268, was observed at a concentration of 100 μmol/L (Sun et al. 2011a, 2011b, 2012a, 2012b, 2013; Liu et al. 2017). In particular, **137** exhibited strong inhibitory activity against MCF-7 and SF-268 cells but moderate activity against NCI-H460 cells. **134** showed moderate inhibitory activity against MCF-7 and SF-268 cells but only weak activity against NCI-H460 cells. However, **152** exhibited strong inhibitory activity across all three tumour cell lines. Furthermore, the IC_50_ values for **152** were determined to be 0.022 μmol/L for MCF-7 cells, 0.073 μmol/L for NCI-H460 cells, and 0.059 μmol/L for SF-268 cells (Sun et al. 2011b). Structure-activity relationship (SAR) analysis revealed the significance of the epoxy structure between C-6 and C-7 in phenochalasin B (**152**) for its potent antitumor activity (Sun et al. 2011b). Compounds **9** and **10** exhibited only weak inhibitory activity against the MCF-7 cell line (Sun et al. 2012b). Compound **90** showed strong inhibitory activity against three tumour cell lines, with low IC_50_ values of 9.2 μmol/L, 4.4 μmol/L, and 9.9 μmol/L for SF-268, MCF-7, and NCI-H460 cell lines, respectively. Whereas **93** exhibited certain cytotoxic selectivity against different tumour cell lines, with the strongest inhibitory effect on MCF-7 with an IC_50_ value of 12.0 μmol/L, followed by SF-268 with an IC_50_ value of 20.5 μmol/L, and the weakest on NCI-H460 with an IC_50_ value of 40.2 μmol/L. It is hypothesised that the ketone group at the C-7 position of diaporthein B (**90**) is necessary for its activity (Sun et al. 2011a). Compound **153** displayed moderate inhibitory activity against SF-268 and MCF-7 cells with IC_50_ values of 35.4 μmol/L and 47.2 μmol/L, respectively (Sun et al. 2013). Scopararanes C and D (**94**–**95**) had moderate inhibitory activity against the MCF-7 cells with IC_50_ values of 35.9 μmol/L and 25.6 μmol/L, respectively. In addition, compounds **88**, **96**, and **98** exhibited weak inhibitory activity against the MCF-7 cell line with IC_50_ values of 60.1–85.5 μmol/L (Sun et al. 2012a). Scopararane I (**103**) showed moderate inhibitory activity across three tumour cell lines, with IC_50_ values of 83.91 μmol/L for MCF-7, 13.59 μmol/L for NCI-H460, and 25.31 μmol/L for SF-268 (Liu et al. 2017).

**Figure 7.**
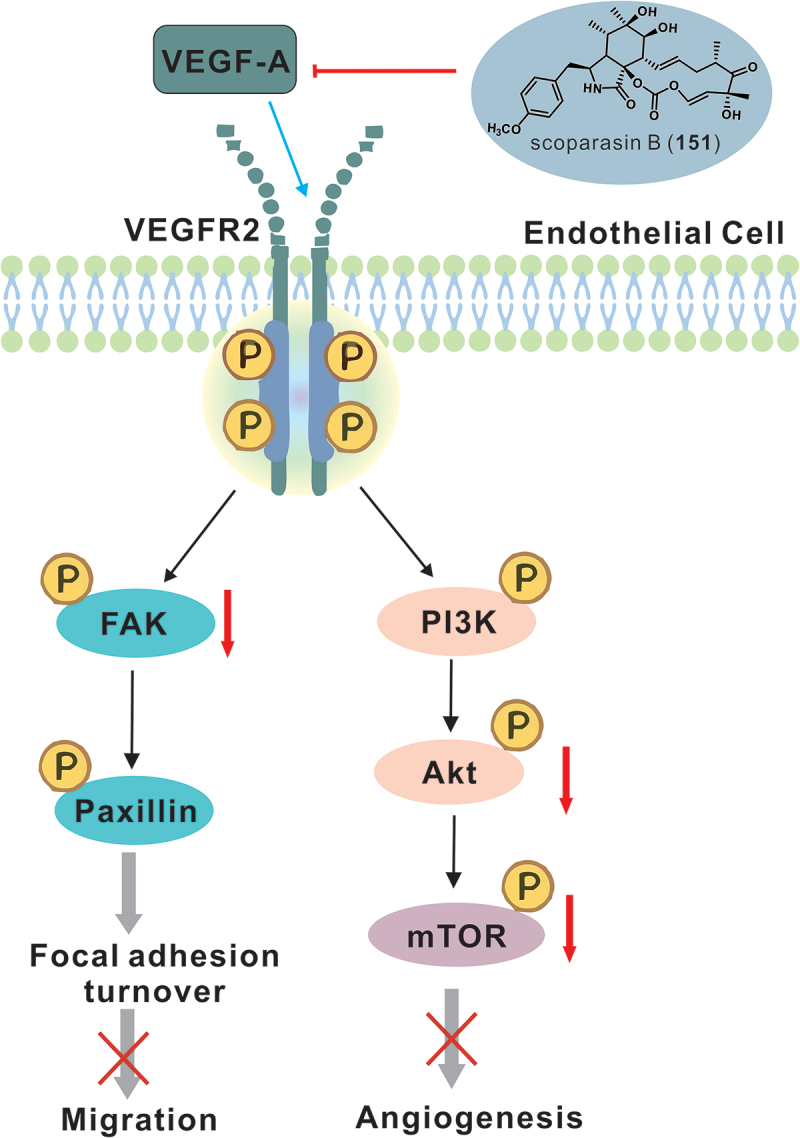
Schematic representation of the inhibitory mechanism of scoparasin B (**151**) against angiogenesis and vasculogenic mimicry in non-small-cell lung cancer.

Compounds **5** and **161** showed weak effects on NCI-H187, MCF-7, and KB cell lines (Isaka et al. 2009, 2011), while **87** and **90** showed potent inhibitory activities, especially against NCI-H187 cells (Isaka et al. 2011). Compounds **144**–**147** showed moderate cytotoxicity against DU145, SW1990, Huh7, and PANC-1 cells, with IC_50_ values below 17.1 μmol/L (Zhang et al. 2019). Similarly, **250** showed moderate inhibitory activity against PANC-1 and SW1990 cells, with IC_50_ values of 13.4 μmol/L and 10.3 μmol/L, respectively (Yu et al. 2020). Compound **152** was found to be highly cytotoxic to KB cells with an IC_50_ of 2.46 μmol/L (Kongprapan et al. 2015). Compound **154** showed relative antitumour activities against several cancer cell lines with IC_50_ values of 9.33 μmol/L for MCF-7, 20.9 μmol/L for NCI-H460, 26.78 μmol/L for Huh-7, and 38.66 μmol/L for SG7901, while compound **151** was specific for the NCI-H460 lung cell line with an IC_50_ of 3.9 μmol/L (Liu et al. 2014). Compounds **148**–**151** showed moderate antiproliferative effects on MCF-7, HeLa, and MDA-MB-231 cell lines, with IC_50_ values ranging from 4.11 to 40.46 μmol/L (Zhang et al. 2021b). Compound **151** has also been found to strongly inhibit angiogenesis and angiogenic mimicry *in vitro* and *ex vivo*, specifically by decreasing VEGF-A levels and inhibiting the VEGF-A/VEGFR2 signalling pathway in non-small-cell lung cancer (Lin et al. 2023) (Figure 7). Compound **159** showed significant cytotoxicity against four cancer cell lines with IC_50_ values of 1.08 μmol/L for A375, 2.25 μmol/L for A549, 1 μmol/L for HepG2, and 3.40 μmol/L for MCF-7 cells (Qi et al. 2015). Compound **150** showed moderate inhibitory activity against these four cell lines, with IC_50_ values ranging from 5.74 to 10.85 μmol/L (Qi et al. 2015). Phenochalasin B (**152**) showed potent antitumour activities against these four cell lines, with IC_50_ values not exceeding 1.8 μmol/L (Qi et al. 2015). It is worth noting that **152** has been widely reported for its broad and excellent antitumour activities (Sun et al. 2011b; Kongprapan et al. 2015; Qi et al. 2015).

Compound **121** effectively inhibited the growth of the HCT-116 cell line with an IC_50_ value of 3.7 μmol/L (Yu et al. 2018a). Compound **141** inhibited the cancer-related protein Mcl-1, which is associated with apoptosis, with an IC_50_ of 11.4 μmol/L (Oh et al. 2010). Compounds **105**, **108**–**112**, **118**–**119**, and **126** were tested for anticancer activities against five human cancer cell lines, HeLa, MCF-7, HCT-116, PANC-1, and SW1990 cells (Yu et al. 2018b). In particular, **108**–**110** showed effective inhibition against these cell lines with IC_50_ values not higher than 6.0 μmol/L, **118**–**119** showed moderate inhibitory activities against these cell lines with IC_50_ values not higher than 13.1 μmol/L, and **105** showed the strongest cytotoxicity against these cell lines with IC_50_ values ranging from 0.3 to 1.8 μmol/L (Yu et al. 2018b). In another experiment, compound **105** showed broad cytotoxicity against U251, SW-1990, SG7901, MCF-7, Huh-cells, Hela, and NCI-H460 cell lines with IC_50_ values ranging from 3.31 to 44.1 μmol/L (Lu et al. 2014), while **8** showed only weak cytotoxicity against the SGC7901 cell line with an IC_50_ value of 39.8 μmol/L (Zhou et al. 2017). SAR analysis suggested that the cyclopropane structure in **105** may be an important feature of its biological activity (Lu et al. 2014). In addition, **93** and **107** exhibited moderate inhibitory activities on the growth of HeLa, MCF-7, K562, and SW1990 cell lines with IC_50_ values ranging from 7.67 to 30.06 μmol/L, whereas **125** showed only weak cytotoxicity against K562 cells with an IC_50_ value of 35.99 μmol/L (Wang et al. 2018). Compound **82** showed moderate antitumour activity against JEKO-1 and HepG2 cell lines with IC_50_ values of 8.4 μmol/L and 28.5 μmol/L respectively, while compound **86** had only a weak effect on the HepG2 cell line with an IC_50_ of 48.4 μmol/L (Wang et al. 2017). Compound **254**, the first demonstrated vincristine of fungal origin, was evaluated for antiproliferative activity against A431, HeLa, and A549 tumour cells with IC_50_ values of 4.8 ± 0.33 μg/mL for A431, 10.0 ± 0.25 μg/mL for HeLa and 14.0 ± 0.17 μg/mL for A459, respectively (Kuriakose et al. 2016). Further studies on the cytotoxicity and apoptosis induction of **254** in A431 cells showed that **254** induced apoptosis of A431 cells through mitochondrial membrane depolarisation, ROS production, and DNA breaks, thus inhibiting the growth and proliferation of A431 cells (Kuriakose et al. 2016).

Eleven compounds including **5**, **87**, **89**–**91**, **150**, **152**–**153**, **157**, **159**, and **161** were tested for their cytotoxic activities against Vero cells (Isaka et al. 2009, 2011; Kongprapan et al. 2015). The results showed that **5**, **153**, and **161** exhibited weak effects with IC_50_ values above 32 μmol/L (Isaka et al. 2009; Kongprapan et al. 2015). Compounds **89** and **91** showed moderate cytotoxicity with IC_50_ values of 10.76 μmol/L and 12.91 μmol/L, respectively (Kongprapan et al. 2015). While **87**, **90**, **150**, **152, 157**, and **159** showed significant cytotoxic activities with IC_50_ values below 2.8 μmol/L (Isaka et al. 2011; Kongprapan et al. 2015).

### 3.2. Antimicrobial activities

A total of 21 compounds were found to have inhibitory activity against bacteria and fungi. Compound **90** possessed antimicrobial activity against *Staphylococcus aureus* ATCC 25923, with a MIC value of 87.8 μmol/L, and compound **151** showed antimicrobial activity against *Microsporum gypseum* SH-MU-4, with a MIC value of 30.3 μmol/L (Pongcharoen et al. 2006). In addition, compound **90** also has been reported to have antimycobacterial activity against *Mycobacterium tuberculosis* H37Ra, with a MIC value of 8.6 μmol/L (Isaka et al. 2011). Compound **121** inhibited the *S. aureus* and *Escherichia coli* with MIC values of both 8 μg/mL meanwhile showed antifungal activity against *Candida parapsilosis*, *C. albicans*, *C. glabrata*, and *C. tropicalis* with MIC values ranging from 8 to 32 μg/mL (Yu et al. 2018a). Additionally, compounds **19**, **193**–**196** displayed antibacterial activity against *S. aureus* and methicillin-resistant *S. aureus* (MRSA) with MIC values under 6.3 μg/mL (Zhang et al. 2021a, 2021b). Antimicrobial activity evaluation based on the disk diffusion method showed that **104** possessed moderate antimicrobial activity against *E. coli*, *Bacillus subtilis*, and *S. aureus* (Lu et al. 2014), whereas compounds **7** and **8** exhibited antimicrobial activities comparable to that of the positive controls (chloramphenicol and ampicillin) against these three bacteria, respectively (Lu et al. 2015; Zhou et al. 2017).

Compound **82** exhibited moderate inhibitory activity against *B. subtilis* CMCC63501 and *B. pumilus* CMCC63202 with IC_50_ values of 18.1 μmol/L and 23.8 μmol/L, respectively (Wang et al. 2017). Compound **172** showed strong antibacterial activity against *Micrococcus lysodeikticus* and *Enterobacter aerogenes* with MIC values of both 3.12 μmol/L (Liao et al. 2017). Compounds **93** and **106** displayed moderate antibacterial activity against *E. coli*, *S. aureus*, and *Vibrio vulnificus* with MIC values ranging from 16–32 μg/mL (Wang et al. 2018). Compound **202** showed moderate antifungal activity against *Athelia rolfsii* with a MIC value of 250 μg/mL (Tanapichatsakul et al. 2020). Compounds **222**–**224** exerted moderate antimicrobial effects on *S. aureus* ATCC 25-923 and vancomycin-resistant enterococci, with MIC values of 16–32 μmol/L (Niu et al. 2017b). The diterpene glycoside analog sordarin, **142**, was a potent antifungal antibiotic that exhibited inhibitory activity against some pathogens (Vicente et al. 2009). Zofimarin (**143**), a sordarin derivative, was also a potent antifungal antibiotic, with particularly strong activity against pathogenic yeasts (Vicente et al. 2009).

### 3.3. Anti-inflammatory activity

Eutypenoid B (**114**) was assessed to have a significant inhibitory effect on the proliferation of splenocytes induced by concanavalin A (ConA) and **114** showed no cytotoxic effect on splenocytes in the concentration range of 1.6 to 40 μmol/L (Zhang et al. 2016). Compounds **167**, **172**, **175**, **179**, and **183** showed immunosuppressive activity against ConA-induced T-cell proliferation, with inhibition rates ranging from 55.8%–68.7% at 5 μg/mL (Yu et al. 2023). Compounds **118**–**119** dose-dependently inhibited the production of TNF-α, an inflammatory factor of the NF-κB pathway, and had a significant inhibitory effect on lipopolysaccharide (LPS)-induced NO production in RAW264.7 macrophages (Yu et al. 2018b). Furthermore, compounds **93**, **123**, and **129** displayed potent inhibition of NO-releasing from the LPS-induced RAW264.7 cells at 10 μmol/L, with a maximum inhibition rate of 89.4% (Ning et al. 2023).

Five eremophilane sesquiterpenes, **30**, **37**–**38**, **51**, and **58**, showed excellent inhibitory activity against the LPS-activated NO production in RAW264.7 macrophage cells at a concentration of 50 μmol/L. Their inhibition was above 77% and their IC_50_ values were less than 24 ± 1 μmol/L (Niu et al. 2018). The SAR analysis suggested that the peroxy group at C-7 of **37**–**38** may be responsible for the significant bioactivity (Niu et al. 2018). Compounds **60**–**61**, **63**, **71**–**76**, **78**–**79**, exhibited noticeable inhibitory effects against the NO production in LPS-induced RAW 264.7 macrophages. Their IC_50_ values ranged from 8.6 ± 1 μmol/L to 18.7 ± 1 μmol/L, with **72** existing as the best activity (Niu et al. 2021). Compounds **171** and **176**, showed moderate inhibitory activities against the NO production in LPS-induced RAW 264.7 macrophages with inhibition rates of 49.0% and 54.9%, respectively, at concentrations of 50.0 μg/mL (Zhang et al. 2022a).

### 3.4. Antiviral activity

Compounds **167** and **172** showed inhibitory activity against respiratory syncytial virus (RSV) with IC_50_ values of 30.25 μmol/L and 72.01 μmol/L, respectively (Liao et al. 2017). The anti-HIV activity of compounds **207**–**219** was evaluated against pNL4.3. Env-. Luc co-transfected 293T cells. Of these thirteen compounds, all except compound **219** showed some extent of inhibition against the cells tested, with compound **211** showing the highest potency (Niu et al. 2017a). In addition, compounds **216** and **229** were found to reactivate latent HIV-1 transcription at 80 μmol/L, suggesting their potential as promising agents for the treatment of HIV-1 infection (Niu et al. 2017a).

## 4. Biosynthetic analyses

### 4.1. *Biosynthetic analyses for pimarane diterpenes from* Eutypella

**Figure 8.**
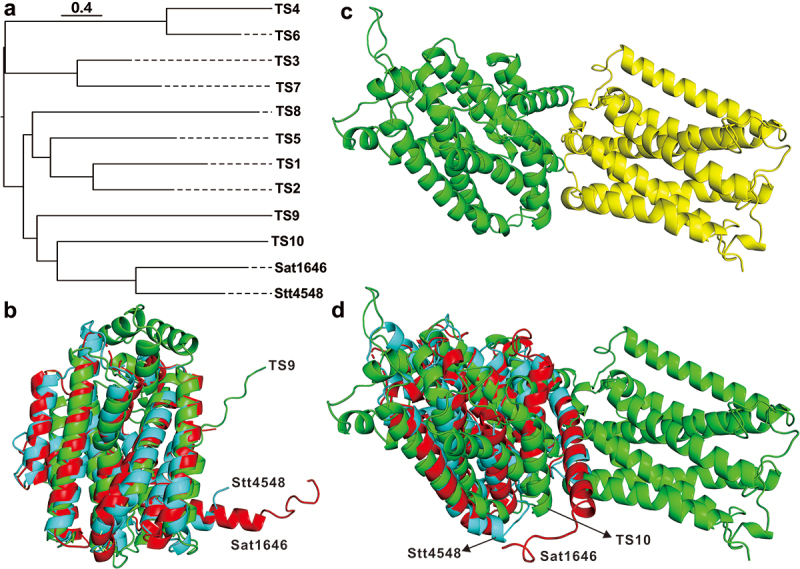
Bioinformatic analysis of terpene-associated enzymes from *Eutypella* sp. D-1. (a) Cluster analysis of terpene-related enzymes with two pimarane diterpene cyclases. (b) Spatial structural comparison of TS9, Stt4548, and Sat1646. (c) Domains of the putative chimeric cyclase TS10. (d) Spatial structural comparison of TS10, Stt4548, and Sat1646.

The pimarane diterpenes, possessing a 6,6,6 tricyclic skeleton, represent a group of natural compounds that are derived from various plant, fungal, and bacterial sources and exhibit a broad range of biological activities (Reveglia et al. 2018; Wang et al. 2018). Despite the structural elucidation of numerous pimarane diterpenes, the identification of bacterial pimarane diterpene cyclases has been limited until recently, with only Stt4548 (PDB ID: 7E4M) and Sat1646 (PDB ID: 7E4O) being discovered (Xing et al. 2021). Among the pimarane diterpene producers in the Diatrypaceae family, six different species of *Eutypella* (Pongcharoen et al. 2006; Isaka et al. 2009, 2011; Sun et al. 2011a, 2012a; Lu et al. 2014; Kongprapan et al. 2015; Liu 2016; Zhang et al. 2016; Liu et al. 2017; Wang et al. 2018; Yu et al. 2018a, 2018b; Ning et al. 2023) and *C. eunomia* var. *eunomia* (Yoshida et al. 2007) have been identified. Using bioinformatics mining of the available genome of *Eutypella* sp. D-1 combined with cluster analysis of terpene synthesis-related enzymes predicted by antiSMASH (Blin et al. 2023) (Table S2), as well as the identification of two pimarane diterpene cyclases (Figure 8(a)), two potential diterpene cyclases, TS9 and TS10, were identified as being responsible for pimarane diterpene biosynthesis in *Eutypella* sp. D-1. Predictions of the three-dimensional (3D) structures of TS9 and TS10 were made using alphafold (Jumper et al. 2021) (Figure 8b,c). The 3D structure of TS9 was found to have some spatial overlap with the crystal structures of the two known cyclases (Figure 8(b)). Interestingly, the predicted 3D structure of TS10 showed two distinct domains, resulting in a high similarity to the identified cyclases (Figure 8(c)). Further analysis of the structural domains revealed that the N-terminal domain of TS10 possessed features of the lycopene cyclase domain, while the C-terminal domain contained an Mg^2+^ binding site and a region characteristic of terpenoid synthase (Figure S1). Consequently, TS10 was proposed as a chimeric terpenoid cyclase. Chimeric terpene cyclases represent a type of bifunctional enzyme commonly found in filamentous fungi and are responsible for the biosynthesis of diterpenes (Minami et al. 2018) and sesquiterpenes (Chen et al. 2021; Zhang et al. 2022c). In particular, a three-dimensional structure comparison revealed a high similarity between the N-terminal domain of TS10 and the crystal structures of Stt4548 and Sat1646 (Figure 8(d)).

### 4.2. *Biosynthetic analyses for scoparasins from* Eutypella

**Figure 9.**
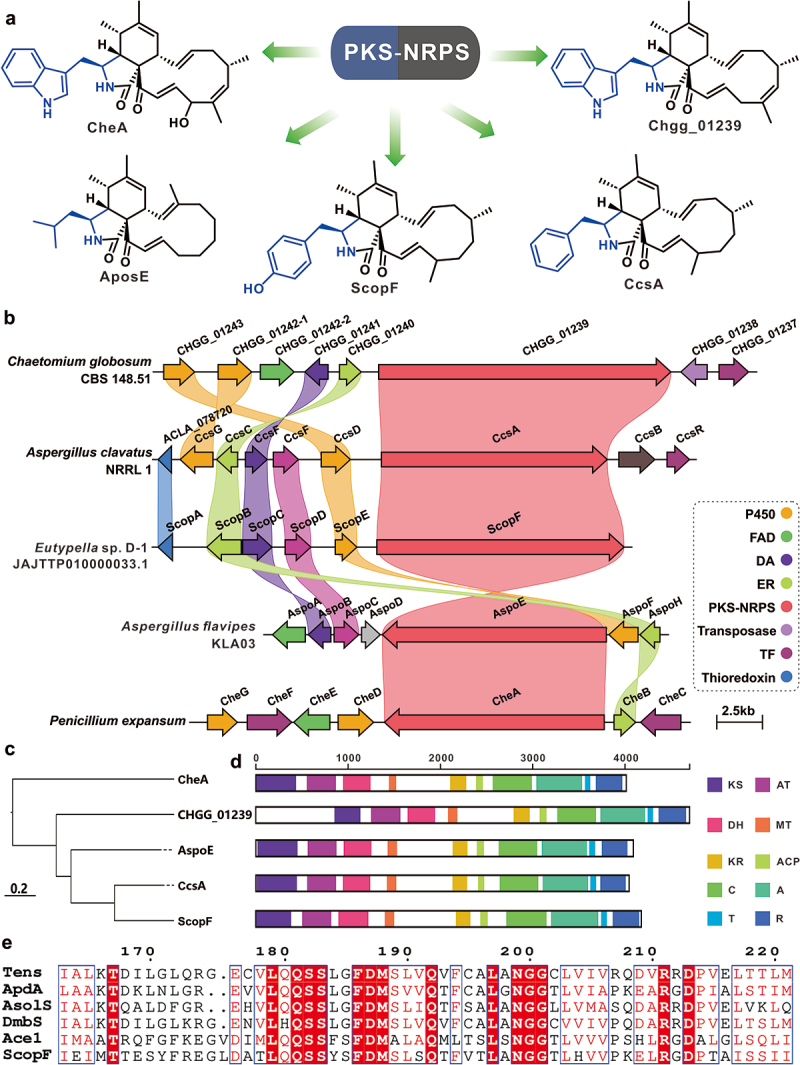
Biosynthesis analysis of cytochalasins. (a) Structural diversity of the cytochalasin family compounds. (b) Comparison of the biosynthetic gene clusters (BGCs) for cytochalasins and the predicted BGC for scoparasins. (c) Cluster analysis of four core enzymes and the predicted PKS-NRPS ScopF. (d) Domain comparison of four core enzymes and the predicted PKS-NRPS ScopF. KS, beta-ketoacyl synthase. AT, acyl transferase. DH, dehydrogenase. MT, methyltransferase. KR, ketoreductase. ACP, acyl carrier protein. C, condensation. A, adenylation. T, thiolation. R, thioester reductase. (e) Conserved sequence analysis of motifs used to recognise amino acids in the A domain.

Since the first discovery of cytochalasins in 1966 (Rothweiler and Tamm 1966), numerous structurally diverse cytochalasins have been isolated from a variety of sources. The structure of cytochalasins is characterised by its tricyclic core structure in which the macrocycle is fused to the bicyclic lactam (“isoindolone”) system. The biosynthesis of four classes of cytochalasins (Schümann and Hertweck 2007; Qiao et al. 2011; Ishiuchi et al. 2013; Zhang et al. 2022b) (Figure 9a), formed with the participation of three amino acids (phenylalanine, tryptophan, and leucine), have been (partially) elucidated. The scoparasin-type cytochalasins are a class of complex macrocyclic compounds involving the formation of tyrosine. In the family Diatrypaceae, strains of the genus *Eutypella* are producers of scoparasins compounds (Pongcharoen et al. 2006; Sun et al. 2011b, 2013; Liu et al. 2014; Kongprapan et al. 2015; Qi et al. 2015; Zhou et al. 2017; Zhang et al. 2021b). With the aid of antiSMASH, a putative scoparasin biosynthetic gene cluster [*scop* BGC (GenBank accession number: PP432626), Figure 9(b), Table S3] was identified in the genome of *Eutypella* sp. D-1 and there is some similarity between *scop* BGC and the identified BGCs from cytochalasins, including *CHGG* BGC (Ishiuchi et al. 2013), *ccs* BGC (Qiao et al. 2011), *aspo* BGC (Zhang et al. 2022b), and *che* BGC (Schümann and Hertweck 2007) (Figure 9(b)). Cluster analysis revealed that ScopF and CcsA have a higher affinity (Figure 9(c)). Comparison of the domain compositions of the five core enzymes showed high consistency in the type and order [ketone synthase(KS)-acyltransferase(AT)-dehydratase(DH)-methyltransferase(MT)-ketoreductase(KR)-acyl carrier protein(ACP)-condensation(C)-adenylation(A)-thiolation(T)-reductase(R)] of these five PKS-NRPS (Figure 9(d)). To further explore the mechanism of tyrosine recognition by the A domain of ScopF, the A domains of identified hybrid enzymes that use tyrosine as a substrate were used for alignment analysis (Figure S2). A conserved region consisting of two motifs, FDMXXXQ and LXNGG (X refers to any amino acid, Figure 9(e)), was proposed to be the key site for tyrosine recognition.

### 4.3. Biosynthetic progresses on aromatic acetylenic compounds

**Figure 10.**
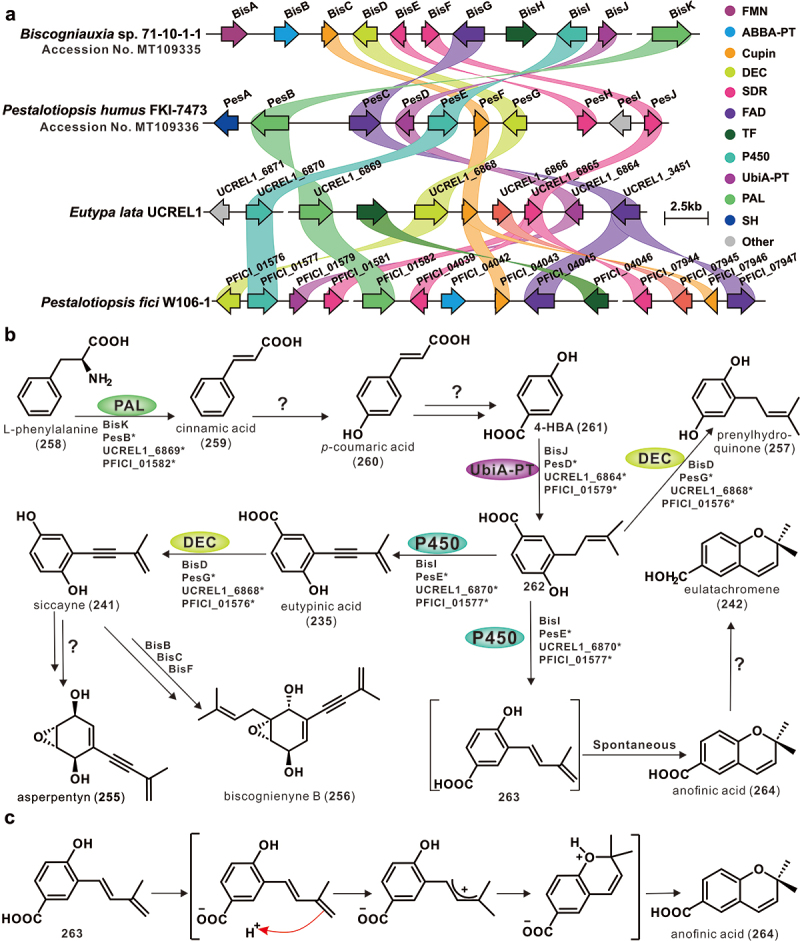
Biosynthesis of aromatic acetylene compounds in filamentous fungi. (a) Comparison of the biosynthetic gene clusters (BGCs) for aromatic acetylene compounds. (b) Biosynthetic pathways for aromatic acetylene compounds. (c) The proposed cyclisation mechanism for the formation of chromene compounds.

Aromatic acetylenic compounds are a class of typically unstable compounds characterised by unique carbon-carbon triple bonds, which are widely present in the metabolites of plants, mosses, fungi, and actinomycetes (Minto and Blacklock 2008; Qi et al. 2023). *Eutypa lata* is the only species within the Diatrypaceae family known to produce aromatic acetylenic compounds (Renaud et al. 1989a; Defrancq et al. 1992; Molyneux et al. 2002). The biosynthesis of these compounds, including asperpentyn (**255**), biscognienyne B (**256**), and prenylhydroquinone (**257**), derived from filamentous fungi, was recently elucidated (Chen et al. 2020; Lv et al. 2020). The BGC responsible for these compounds in *E. lata* UCREL1 has also been predicted (Chen et al. 2020). In addition to homologous genes with high identities on the BGC, each BGC contains its unique genes (Figure 10(a)). The primary metabolite, L-phenylalanine (**258**), is proposed as the initial substrate in this pathway, which is converted by phenylalanine ammonia-lyase to cinnamic acid (**259**), and then further transformed into *p*-coumaric acid (**260**) and *p*-hydroxybenzoic acid (4-HBA, **261**). Under the modification of UbiA-PT, 4-HBA (**261**) forms an indispensable intermediate, **262**. This intermediate, **262**, undergoes a specific P450 modification to form an alkynyl-containing compound, **235**, and then transformed into a decarboxylated product, **241**. The intermediate **241** enter the post-modification pathways leading to asperpentyns compounds and biscognienynes compounds, respectively. Intermediate **262** is crucial as it not only leads to the formation of byproduct **257** but also generates compound **242** with a chromene skeleton. **263** is a proposed byproduct of intermediate **262** through P450 modification, and it is speculated that this unstable intermediate spontaneously converts to chromene compound **264** (Figure 10(b)). The proposed cyclisation mechanism is depicted in Figure 10(c).

## 5. Discussions, conclusions, and perspectives

Since the first publication in 1989, a total of 254 secondary metabolites have been isolated and identified from species within the Diatrypaceae family. These compounds possess some hidden features and patterns that may not be readily apparent. Diterpenoids derived from Diatrypaceae are exclusive of the pimarane diterpenes and originate from species in the *Eutypella* genus (Pongcharoen et al. 2006; Isaka et al. 2011; Sun et al. 2012a; Lu et al. 2014; Zhang et al. 2016; Liu et al. 2017; Wang et al. 2018; Yu et al. 2018a, 2018b; Zhang et al. 2019; Ning et al. 2023). It is deduced that the genes encoding the cyclases responsible for the cyclisation of pimarane terpenoids are conserved exclusively in the *Eutypella* genus within the Diatrypaceae family. Pimarane diterpenoids are significant tricyclic diterpenoids and can be classified into four types, pimarane, isopimarane, *ent*-pimarane, and *ent*-isopimarane, based on different chiral centres (Wang et al. 2018). The majority of the diterpenoids summarised in this review belong to the isopimarane type, with a small portion falling into the pimarane type and other categories. These diterpenoids exhibit a wide range of biological activities, including anticancer, antimicrobial, and anti-inflammatory properties. The cytochalasin compound phenochalasin B (**152**) shows broad-spectrum and potent anticancer activity, displaying stronger inhibitory effects on tumour cell lines MCF-7, NCI-H460, and SF-268 than the positive control drug cisplatin (Sun et al. 2011b; Kongprapan et al. 2015; Qi et al. 2015). Given the potent activity demonstrated by **152**, this compound holds promise as a candidate or lead compound for the development of novel anticancer agents. Eutypellazines A–L (**207**–**218**), a novel series of thiodiketopiperazine alkaloids, exhibit excellent anti-HIV activity and have potential applications in the development of new anti-HIV drugs (Niu et al. 2017a). In particular, eutypellazine J (**216**) and epicoccin A (**229**) can reactivate latent HIV-1 transcription, and thus contribute to the complete eradication of the virus (Niu et al. 2017a). This is a rare occurrence among natural compounds.

The study of toxic compounds produced by pathogenic organisms is often specifically linked to natural product chemistry, with many of the most damaging pathogens producing a diverse range of secondary metabolites, such as *Colletotrichum* (Moraga et al. 2019) and *Fusarium* (Perincherry et al. 2019; Lin et al. 2023). Members of the Diatrypaceae family are widespread microorganisms in terrestrial and marine environments worldwide, and some are serious plant pathogens. Within the Diatrypaceae family, *E. lata* is a typical representative that produces aromatic alkynyl compounds and their derivatives that are widely believed to be involved in *E. lata*-induced grapevine wilt (Tey-Rulh et al. 1991; Molyneux et al. 2002). As far as aromatic alkynyl compounds are concerned, they are a class of toxin molecules widely present in filamentous fungi, including *Aspergillus*, *Pestalotiopsis*, *Biscogniauxia*, and *Eutypa*, and their biosynthetic pathways are both related and unique (Chen et al. 2020; Lv et al. 2020) (Figure 10(a)). The biosynthetic elucidation of such compounds may facilitate the investigation of their pathogenic mechanisms.

Filamentous fungi are a rich source of natural products, with classic examples including the antibiotic penicillin, the cholesterol-lowering drug lovastatin, and the anti-cancer agent griseofulvin, all derived from filamentous fungi. Advances in sequencing technologies have led to the publication of numerous fungal genomes, coupled with continuous improvements in bioinformatics prediction tools, increasing the popularity of research on natural product biosynthesis in filamentous fungi (Yuan et al. 2022). Functional gene elements discovered through biosynthesis studies provide critical support for synthetic biology approaches aimed at the production of valuable natural products from filamentous fungi. In addition to the traditional chassises *Aspergillus nidulans* (Chiang et al. 2021) and *A. oryzae* (Qi et al. 2022), other important filamentous fungi such as *A. terreus*, *Candida* sp., and *Rhizopus* sp. have become or are becoming important emerging chassis (Ding and Ye 2023). Given the abundant secondary metabolite synthesis capacity of species within the Diatrypaceae family, chassis design and modification of related species within the Diatrypaceae family hold promising prospects for exploration.

In conclusion, this comprehensive review investigated the metabolites from the Diatrypaceae family and collected and summarised 254 natural compounds with diverse structures. Many of these metabolites exhibit significant biological activities, such as anti-cancer, cytotoxic, antimicrobial, anti-inflammatory, and antiviral properties. The review provides an in-depth analysis of the biosynthesis of pimarane diterpenes and scoparasin-type cytochalasins. Furthermore, a comparative analysis is conducted on the biosynthesis of identified aromatic acetylene compounds and the speculated biosynthesis of aromatic acetylene compounds in the Diatrypaceae family. This thorough review not only enhances our understanding of the natural product chemistry, biological activities, and biosynthesis of secondary metabolites from the Diatrypaceae family but also promotes the biotechnological development of these important bioactive compounds and potential strains.

## Supplementary Material

Supplemental Material

## Data Availability

The datasets used and/or analysed during the current study are available from the corresponding author upon reasonable request. The tool Synthaser was used to analyse the domain characteristics of NRPS-PKS hybrid synthases (Gilchrist and Chooi 2021b). BGC comparisons and their visualisation were performed using Clinker (Gilchrist and Chooi 2021a). Three-dimensional structure modelling was performed using a locally installed AlphaFold database (Jumper et al. 2021) (https://alphafold.com). Evolutionary tree-based cluster analysis was implemented using the Cluster Omega website (https://www.ebi.ac.uk/Tools/msa/clustalo/, accessed 25 December 2023). Biosynthetic gene cluster prediction was performed using antiSMASH (https://github.com/antismash/antismash).
