# Supplementary material for: Chemical structures, biological activities, and biosynthetic analysis of secondary metabolites of the Diatrypaceae family: A comprehensive review
Source: Mycology. 2024 Apr 24;15(3):322–44. doi: 10.1080/21501203.2024.2341648 (PMC11376284; doi:10.1080/21501203.2024.2341648)
Supplement: Supplemental Material [file TMYC_A_2341648_SM9684.docx]

# Table S1. Compounds 1–254 from the family Diatrypaceae and their biological activities.

| **No.** | **Name** | **Producer** | **Isolated source** | **Biological activity** | **Reference** |
| --- | --- | --- | --- | --- | --- |
| **1** | (3*S*,3a*R*,7a*S*)-3a,4,5,7a-tetrahydro-3,6-dimethylbenzofuran-2(3*H*)-one | *Eutypella scoparia*. FS26 | Fermentation broth |  | (Sun et al. 2011b, 2012a) |
| **2** | eutypellol B | *Eutypella scoparia* FS46 | Culture broth |  | (Liu et al. 2017a) |
| **3** | 2-(2-hydroxy-4-methylcyclohex-3-enyl) propanoic acid | *Eutypella scoparia* FS46 | Culture broth |  | (Liu et al. 2017a) |
| **4** | 2,9-epoxy-p-menth-6-en-9-ol | *Eutypella scoparia* FS46 | Culture broth |  | (Liu et al. 2017a) |
| **5** | *ent*-4(15)-eudesmen-11-ol-1-one | *Eutypella* sp. BCC 13199;  *Eutypella scoparia* SCBG-8;  *Eutypella scoparia* 1-15 | Fermentation broth; solid medium | Antitumor activity;  cytotoxic activity against Vero cells | (Isaka et al. 2009; Qi et al. 2015; Zhang et al. 2021a) |
| **6** | *ent*-4(15)-eudesmen-1*α*,11-diol | *Eutypella* sp. BCC 13199 | Fermentation broth |  | (Isaka et al. 2009) |
| **7** | *eut*-Guaiane sesquiterpene | *Eutypella* sp. D-1 | Culture broth | Antibacterial activity | (Lu et al. 2015) |
| **8** | *eut*-Guaiane sesquiterpene | *Eutypella* sp. D-1;  *Eutypella scoparia* SCBG-8 | Culture broth | Antitumor activity;  antibacterial activity | (Zhou et al. 2017; Zhang et al. 2021a) |
| **9** | 3,7,10-trihydroxy-6,11-cyclofarnes-1-ene | *Eutypella scoparia* FS26 | Fermentation broth | Antitumor activity | (Sun et al. 2012a) |
| **10** | 8-(hydroxymethyl)-1-(1-hydroxy-1-methylethyl)-4-methylspiro[4.5]dec-8-en-7-ol | *Eutypella scoparia* FS26 | Fermentation broth | Antitumor activity | (Sun et al. 2012a) |
| **11** | scopararane C | *Eutypella scoparia* 1-15 | Solid medium |  | (Qi et al. 2015) |
| **12** | eutypellol A | *Eutypella scoparia* FS46 | Culture broth |  | (Liu et al. 2017a) |
| **13** | eutyscoparin A | *Eutypella scoparia* SCBG-8 | Fermentation broth |  | (Zhang et al. 2021a) |
| **14** | eutyscoparin B | *Eutypella scoparia* SCBG-8 | Fermentation broth |  | (Zhang et al. 2021a) |
| **15** | eutyscoparin C | *Eutypella scoparia* SCBG-8 | Fermentation broth |  | (Zhang et al. 2021a) |
| **16** | eutyscoparin D | *Eutypella scoparia* SCBG-8 | Fermentation broth |  | (Zhang et al. 2021a) |
| **17** | eutyscoparin E | *Eutypella scoparia* SCBG-8 | Fermentation broth |  | (Zhang et al. 2021a) |
| **18** | eutyscoparin F | *Eutypella scoparia* SCBG-8 | Fermentation broth |  | (Zhang et al. 2021a) |
| **19** | eutyscoparin G | *Eutypella scoparia* SCBG-8 | Fermentation broth | Antibacterial activity;  anti-MRSA activity | (Zhang et al. 2021a) |
| **20** | eutyscoparin H | *Eutypella scoparia* SCBG-8 | Fermentation broth |  | (Zhang et al. 2021a) |
| **21** | *rel*-(3*S*,6*S*,7*R*,10*R*)-7,10-epoxy-3,7,11-trimethyldodec-1-ene-3,6,11-triol | *Eutypella scoparia* FS26 | Fermentation broth |  | (Sun et al. 2011b, 2012a) |
| **22** | pterocarpol | *Eutypella scoparia* FS46;  *Eutypella scoparia* SCBG-8 | Culture broth |  | (Liu et al. 2017a; Zhang et al. 2021a) |
| **23** | isopterocarpolone | *Eutypella scoparia* FS46 | Culture broth |  | (Liu et al. 2017a) |
| **24** | pterocarpol monoacetate | *Eutypella scoparia* FS46 | Culture broth |  | (Liu et al. 2017a) |
| **25** | carissone | *Eutypella scoparia* FS46;  *Eutypella scoparia* SCBG-8 | Culture broth |  | (Liu et al. 2017a; Zhang et al. 2021a) |
| **26** | 5*α*-hydroxy-*β*-eudesmol | *Eutypella scoparia* FS46 | Culture broth |  | (Liu et al. 2017a) |
| **27** | (3*β*)-eudesm-4(14)-ene-3,11-diol | *Eutypella scoparia* FS46 | Culture broth |  | (Liu et al. 2017a) |
| **28** | ambrosanoli-10(14)-en-11,12-diol | *Eutypella scoparia* FS46 | Culture broth |  | (Liu et al. 2017a) |
| **29** | eutyperemophilane A | *Eutypella* sp. MCCC 3A00281 | Modified rice medium |  | (Niu et al. 2018) |
| **30** | eutyperemophilane B | *Eutypella* sp. MCCC 3A00281 | Modified rice medium | Anti-inflammatory activity | (Niu et al. 2018) |
| **31** | eutyperemophilane C | *Eutypella* sp. MCCC 3A00281 | Modified rice medium |  | (Niu et al. 2018) |
| **32** | eutyperemophilane D | *Eutypella* sp. MCCC 3A00281 | Modified rice medium |  | (Niu et al. 2018) |
| **33** | eutyperemophilane E | *Eutypella* sp. MCCC 3A00281 | Modified rice medium |  | (Niu et al. 2018) |
| **34** | eutyperemophilane F | *Eutypella* sp. MCCC 3A00281 | Modified rice medium |  | (Niu et al. 2018) |
| **35** | eutyperemophilane G | *Eutypella* sp. MCCC 3A00281 | Modified rice medium |  | (Niu et al. 2018) |
| **36** | eutyperemophilane H | *Eutypella* sp. MCCC 3A00281 | Modified rice medium |  | (Niu et al. 2018) |
| **37** | eutyperemophilane I | *Eutypella* sp. MCCC 3A00281 | Modified rice medium | Anti-inflammatory activity | (Niu et al. 2018) |
| **38** | eutyperemophilane J | *Eutypella* sp. MCCC 3A00281 | Modified rice medium | Anti-inflammatory activity | (Niu et al. 2018) |
| **39** | eutyperemophilane K | *Eutypella* sp. MCCC 3A00281 | Modified rice medium |  | (Niu et al. 2018) |
| **40** | eutyperemophilane L | *Eutypella* sp. MCCC 3A00281 | Modified rice medium |  | (Niu et al. 2018) |
| **41** | eutyperemophilane M | *Eutypella* sp. MCCC 3A00281 | Modified rice medium |  | (Niu et al. 2018) |
| **42** | eutyperemophilane N | *Eutypella* sp. MCCC 3A00281 | Modified rice medium |  | (Niu et al. 2018) |
| **43** | eutyperemophilane O | *Eutypella* sp. MCCC 3A00281 | Modified rice medium |  | (Niu et al. 2018) |
| **44** | eutyperemophilane P | *Eutypella* sp. MCCC 3A00281 | Modified rice medium |  | (Niu et al. 2018) |
| **45** | eutyperemophilane Q | *Eutypella* sp. MCCC 3A00281 | Modified rice medium |  | (Niu et al. 2018) |
| **46** | eutyperemophilane R | *Eutypella* sp. MCCC 3A00281 | Modified rice medium |  | (Niu et al. 2018) |
| **47** | eutyperemophilane S | *Eutypella* sp. MCCC 3A00281 | Modified rice medium |  | (Niu et al. 2018) |
| **48** | eutyperemophilane T | *Eutypella* sp. MCCC 3A00281 | Modified rice medium |  | (Niu et al. 2018) |
| **49** | eutyperemophilane U | *Eutypella* sp. MCCC 3A00281 | Modified rice medium |  | (Niu et al. 2018) |
| **50** | eutyperemophilane V | *Eutypella* sp. MCCC 3A00281 | Modified rice medium |  | (Niu et al. 2018) |
| **51** | eutyperemophilane W | *Eutypella* sp. MCCC 3A00281 | Modified rice medium | Anti-inflammatory activity | (Niu et al. 2018) |
| **52** | eutyperemophilane X | *Eutypella* sp. MCCC 3A00281 | Modified rice medium |  | (Niu et al. 2018) |
| **53** | eutyperemophilane Y | *Eutypella* sp. MCCC 3A00281 | Modified rice medium |  | (Niu et al. 2018) |
| **54** | eutyperemophilane Z | *Eutypella* sp. MCCC 3A00281 | Modified rice medium |  | (Niu et al. 2018) |
| **55** | guignarderemophilane E | *Eutypella* sp. MCCC 3A00281 | Modified rice medium |  | (Niu et al. 2018) |
| **56** | 1*α*-hydroxyeremophila-9,11-dien-8-one | *Eutypella* sp. MCCC 3A00281 | Modified rice medium |  | (Niu et al. 2018) |
| **57** | 1*β*-hydroxyeremophila-7(11),9-dien-8-one | *Eutypella* sp. MCCC 3A00281 | Modified rice medium |  | (Niu et al. 2018) |
| **58** | eremophilane lactam | *Eutypella* sp. MCCC 3A00281 | Modified rice medium | Anti-inflammatory activity | (Niu et al. 2018) |
| **59** | eutypeterpene A | *Eutypella* sp. MCCC 3A00281 | Modified rice medium |  | (Niu et al. 2021) |
| **60** | eutypeterpene B | *Eutypella* sp. MCCC 3A00281 | Modified rice medium | Anti-inflammatory activity | (Niu et al. 2021) |
| **61** | eutypeterpene C | *Eutypella* sp. MCCC 3A00281 | Modified rice medium | Anti-inflammatory activity | (Niu et al. 2021) |
| **62** | eutypeterpene D | *Eutypella* sp. MCCC 3A00281 | Modified rice medium |  | (Niu et al. 2021) |
| **63** | eutypeterpene E | *Eutypella* sp. MCCC 3A00281 | Modified rice medium | Anti-inflammatory activity | (Niu et al. 2021) |
| **64** | eutypeterpene F | *Eutypella* sp. MCCC 3A00281 | Modified rice medium |  | (Niu et al. 2021) |
| **65** | eutypeterpene G | *Eutypella* sp. MCCC 3A00281 | Modified rice medium |  | (Niu et al. 2021) |
| **66** | eutypeterpene H | *Eutypella* sp. MCCC 3A00281 | Modified rice medium |  | (Niu et al. 2021) |
| **67** | eutypeterpene I | *Eutypella* sp. MCCC 3A00281 | Modified rice medium |  | (Niu et al. 2021) |
| **68** | eutypeterpene J | *Eutypella* sp. MCCC 3A00281 | Modified rice medium |  | (Niu et al. 2021) |
| **69** | eutypeterpene K | *Eutypella* sp. MCCC 3A00281 | Modified rice medium |  | (Niu et al. 2021) |
| **70** | eutypeterpene L | *Eutypella* sp. MCCC 3A00281 | Modified rice medium |  | (Niu et al. 2021) |
| **71** | eutypeterpene M | *Eutypella* sp. MCCC 3A00281 | Modified rice medium | Anti-inflammatory activity | (Niu et al. 2021) |
| **72** | eutypeterpene N | *Eutypella* sp. MCCC 3A00281 | Modified rice medium | Anti-inflammatory activity | (Niu et al. 2021) |
| **73** | eutypeterpene O | *Eutypella* sp. MCCC 3A00281 | Modified rice medium | Anti-inflammatory activity | (Niu et al. 2021) |
| **74** | eutypeterpene P | *Eutypella* sp. MCCC 3A00281 | Modified rice medium | Anti-inflammatory activity | (Niu et al. 2021) |
| **75** | eutypeterpene Q | *Eutypella* sp. MCCC 3A00281 | Modified rice medium | Anti-inflammatory activity | (Niu et al. 2021) |
| **76** | xylariterpenoid A | *Eutypella* sp. MCCC 3A00281 | Modified rice medium | Anti-inflammatory activity | (Niu et al. 2021) |
| **77** | xylariterpenoid B | *Eutypella* sp. MCCC 3A00281 | Modified rice medium |  | (Niu et al. 2021) |
| **78** | eudesma-3-en-11,15-diol | *Eutypella* sp. MCCC 3A00281 | Modified rice medium | Anti-inflammatory activity | (Niu et al. 2021) |
| **79** | eudesma-4-en-11,15-diol | *Eutypella* sp. MCCC 3A00281 | Modified rice medium | Anti-inflammatory activity | (Niu et al. 2021) |
| **80** | (11*R*)-2,11,12-trihydroxy-*β*-selinene | *Eutypella* sp. ZZ2 | Rice medium |  | (Liao et al. 2017) |
| **81** | (11*S*)-2,11,12-trihydroxy-*β*-selinene | *Eutypella* sp. ZZ2 | Rice medium |  | (Liao et al. 2017) |
| **82** | 13-Hydroxy-3,8,7(11)-eudesmatrien-12,8-olide | *Eutypella* sp. 1-15 | Rice medium | Antitumor activity;  antibacterial activity | (Wang et al. 2017) |
| **83** | 13-Hydroxy-3,5,8,7(11)-eudesmatetraen-12,8-olide | *Eutypella* sp. 1-15 | Rice medium |  | (Wang et al. 2017) |
| **84** | 2-One-13-hydroxy-3,5,8,7(11)-eudesmatetraen-12,8-olide | *Eutypella* sp. 1-15 | Rice medium |  | (Wang et al. 2017) |
| **85** | 8,13-Dihydroxy-3,7(11)-eudesmadien-12,8-olide | *Eutypella* sp. 1-15 | Rice medium |  | (Wang et al. 2017) |
| **86** | 13-Hydroxy-3,7(11)-eudesmadien-12,8-olide | *Eutypella* sp. 1-15 | Rice medium | Antitumor activity | (Wang et al. 2017) |
| **87** | scopararane A | *Eutypella scoparia* PSU-D44;  *Eutypella* sp. BCC 13199;  *Eutypella scoparia* PSU-H267  *Cryptosphaeria eunomia* var*. eunomia* | Fermentation broth | Antitumor activity;  cytotoxic activities against Vero cells | (Pongcharoen et al. 2006; Yoshida et al. 2007; Isaka et al. 2009; Kongprapan et al. 2015) |
| **88** | scopararane B | *Eutypella scoparia* PSU-D44;  *Eutypella scoparia* (Schw.) Ell. et Ev. | Fermentation broth | Antitumor activity | (Pongcharoen et al. 2006; Sun et al. 2011a) |
| **89** | diaporthein A | *Eutypella scoparia* PSU-D44;  *Eutypella scoparia* (Schw.) Ell. et Ev.;  *Eutypella* sp. FS46;  *Eutypella scoparia* PSU-H267;  *Cryptosphaeria eunomia* var*. eunomia* | Fermentation broth | Cytotoxic activities against Vero cells | (Pongcharoen et al. 2006; Yoshida et al. 2007; Sun et al. 2011a; Kongprapan et al. 2015; Liu et al. 2017b) |
| **90** | diaporthein B | *Eutypella scoparia* PSU-D44;  *Eutypella scoparia* (Schw.) Ell. et Ev.;  *Eutypella* sp. BCC 13199;  *Eutypella* sp. FS46;  *Eutypella scoparia* PSU-H267;  *Cryptosphaeria eunomia* var*. eunomia* | Fermentation broth | Antitumor activity;  cytotoxic activities against Vero cells;  antibacterial activity | (Pongcharoen et al. 2006; Yoshida et al. 2007; Isaka et al. 2009; Sun et al. 2011a; Kongprapan et al. 2015; Liu et al. 2017b) |
| **91** | 11-deoxydiaporthein A | *Cryptosphaeria eunomia* var*. eunomia*;  *Eutypella scoparia* (Schw.) Ell. et Ev.;  *Eutypella* sp. FS46;  *Eutypella scoparia* PSU-H267 | Cultrure broth and mycelium | Cytotoxic activities against Vero cells | (Yoshida et al. 2007; Sun et al. 2011a; Kongprapan et al. 2015; Liu et al. 2017b) |
| **92** | isopimara-8(14),15-diene | *Eutypella scoparia* (Schw.) Ell. et Ev. | Cultrure broth and mycelium |  | (Sun et al. 2011a) |
| **93** | libertellenone A | *Eutypella scoparia* (Schw.) Ell. et Ev.;  *Eutypella* sp. FS46;  *Eutypella* sp. D-1 | Cultrure broth and mycelium | Antitumor activity;  antibacterial activity;  anti-inflammatory activity | (Sun et al. 2011a; Lu et al. 2014; Liu et al. 2017b; Wang et al. 2018; Ning et al. 2023) |
| **94** | scopararane C | *Eutypella scoparia* FS26;  *Eutypella* sp. FS46 | Culture broth | Antitumor activity | (Sun et al. 2012b; Liu et al. 2017b) |
| **95** | scopararane D | *Eutypella scoparia* FS26 | Culture broth | Antitumor activity | (Sun et al. 2012b) |
| **96** | scopararane E | *Eutypella scoparia* FS26 | Culture broth | Antitumor activity | (Sun et al. 2012b) |
| **97** | scopararane F | *Eutypella scoparia* FS26 | Culture broth |  | (Sun et al. 2012b) |
| **98** | scopararane G | *Eutypella scoparia* FS26 | Culture broth | Antitumor activity | (Sun et al. 2012b) |
| **99** | eutypellone A | *Eutypella* sp. BCC 13199;  *Eutypella* sp. D-1 | Culture broth |  | (Isaka et al. 2011; Wang et al. 2018; Ning et al. 2023) |
| **100** | eutypellone B | *Eutypella* sp. BCC 13199 | Culture broth |  | (Isaka et al. 2011) |
| **101** | libertellenone C | *Eutypella* sp. BCC 13199;  *Eutypella* sp. D-1 | Culture broth |  | (Isaka et al. 2009, 2011; Lu et al. 2014; Wang et al. 2018) |
| **102** | scopararane H | *Eutypella* sp. FS46 | Culture broth |  | (Liu et al. 2017b) |
| **103** | scopararane I | *Eutypella* sp. FS46 | Culture broth | Antitumor activity | (Liu et al. 2017b) |
| **104** | libertellenone G | *Eutypella* sp. D-1 | Fermentation broth | Antibacterial activity | (Lu et al. 2014) |
| **105** | libertellenone H | *Eutypella* sp. D-1 | Fermentation broth | Antitumor activity | (Lu et al. 2014; Yu et al. 2018b) |
| **106** | libertellenone M | *Eutypella* sp. D-1 | Fermentation broth | Antibacterial activity | (Wang et al. 2018) |
| **107** | libertellenone N | *Eutypella* sp. D-1 | Fermentation broth | Antitumor activity | (Wang et al. 2018) |
| **108** | libertellenone O | *Eutypella* sp. D-1 | Fermentation broth | Antitumor activity | (Yu et al. 2018b) |
| **109** | libertellenone P | *Eutypella* sp. D-1 | Fermentation broth | Antitumor activity | (Yu et al. 2018b) |
| **110** | libertellenone Q | *Eutypella* sp. D-1 | Fermentation broth | Antitumor activity | (Yu et al. 2018b) |
| **111** | libertellenone R | *Eutypella* sp. D-1 | Fermentation broth | Antitumor activity | (Yu et al. 2018b; Ning et al. 2023) |
| **112** | libertellenone S | *Eutypella* sp. D-1 | Fermentation broth | Antitumor activity | (Yu et al. 2018b) |
| **113** | eutypenoid A | *Eutypella* sp. D-1 | Fermentation broth |  | (Liu 2016; Zhang et al. 2016) |
| **114** | eutypenoid B | *Eutypella* sp. D-1 | Fermentation broth | Anti-inflammatory activity | (Zhang et al. 2016) |
| **115** | eutypenoid C | *Eutypella* sp. D-1 | Fermentation broth |  | (Zhang et al. 2016; Yu et al. 2018a) |
| **116** | eutypenoid D | *Eutypella* sp. D-1 | Fermentation broth |  | (Liu 2016) |
| **117** | eutypenoid E | *Eutypella* sp. D-1 | Fermentation broth |  | (Liu 2016) |
| **118** | eutypellenone A | *Eutypella* sp. D-1 | Fermentation broth | Antitumor activity;  anti-inflammatory activity | (Yu et al. 2018b) |
| **119** | eutypellenone B | *Eutypella* sp. D-1 | Fermentation broth | Antitumor activity;  anti-inflammatory activity | (Yu et al. 2018b) |
| **120** | eutypellenoid A | *Eutypella* sp. D-1 | Fermentation broth |  | (Yu et al. 2018a) |
| **121** | eutypellenoid B | *Eutypella* sp. D-1 | Fermentation broth | Antitumor activity;  antibacterial activity；  antifungal activity | (Yu et al. 2018a) |
| **122** | eutypellenoid C | *Eutypella* sp. D-1 | Fermentation broth |  | (Yu et al. 2018a) |
| **123** | libertellenone B | *Eutypella* sp. D-1 | Fermentation broth | Anti-inflammatory activity | (Liu 2016; Wang et al. 2018; Ning et al. 2023) |
| **124** | dehydroabietic acid | *Eutypella* sp. D-1 | Fermentation broth |  | (Liu 2016) |
| **125** | kaempulchraol W | *Eutypella* sp. D-1 | Fermentation broth | Antitumor activity | (Wang et al. 2018) |
| **126** | libertellenone L | *Eutypella* sp. D-1 | Fermentation broth | Antitumor activity | (Yu et al. 2018b) |
| **127** | eutypellenone F | *Eutypella* sp. D-1 | Culture broth |  | (Ning et al. 2023) |
| **128** | libertellenone Y | *Eutypella* sp. D-1 | Culture broth |  | (Ning et al. 2023) |
| **129** | libertellenone Z | *Eutypella* sp. D-1 | Culture broth | Anti-inflammatory activity | (Ning et al. 2023) |
| **130** | (22*E*,24*R*)-ergosta-4,6,8(14)-22-tetraen-3-one | *Eutypella scoparia* (Schw.) Ell. et Ev. | Fermentation broth |  | (Sun et al. 2011a) |
| **131** | ergosterol | *Eutypella scoparia* (Schw.) Ell. et Ev.;  *Eutypella* sp. D-1;  *Eutypa* sp.(#424) | Fermentation broth |  | (Lin et al. 2002; Sun et al. 2011a; Liu 2016) |
| **132** | ergosterol peroxide | *Eutypella scoparia* (Schw.) Ell. et Ev. | Fermentation broth |  | (Sun et al. 2011a) |
| **133** | cerevisterol | *Eutypella scoparia* (Schw.) Ell. et Ev. | Fermentation broth |  | (Sun et al. 2011a) |
| **134** | tuberoside | *Eutypella scoparia* FS26 | Fermentation broth | Antitumor activity | (Sun et al. 2011b) |
| **135** | eutyscoparene A | *Eutypella scoparia* SCBG-8 | Fermentation broth |  | (Zhang et al. 2021a) |
| **136** | eutyscoparene B | *Eutypella scoparia* SCBG-8 | Fermentation broth |  | (Zhang et al. 2021a) |
| **137** | euphorbol | *Eutypella scoparia* FS26 | Fermentation broth | Antitumor activity | (Sun et al. 2011b, 2012a) |
| **138** | C(24)-epimeric mixtures of (22*E*)-ergosta-4,6,8(14)-22-tetraen-3-one | *Eutypella scoparia* SCBG-8 | Fermentation broth |  | (Zhang et al. 2021a) |
| **139** | *β*-sitosterol | *Eutypella scoparia* SCBG-8 | Fermentation broth |  | (Zhang et al. 2021a) |
| **140** | squalene | *Eutypella scoparia* SCBG-8 | Fermentation broth |  | (Zhang et al. 2021a) |
| **141** | cryptosphaerolide | *Cryptosphaeria* sp. CNL-523 | Saline fermentation | Antitumor activity | (Oh et al. 2010) |
| **142** | sordarin | *Diatrype stigma* |  | Antifungal activity | (Vicente et al. 2009) |
| **143** | zofimarin | *Eutypa tetragona* |  | Antifungal activity | (Vicente et al. 2009) |
| **144** | eutypellacytosporin A | *Eutypella* sp. D-1 | Rice medium | Antitumor activity | (Zhang et al. 2019) |
| **145** | eutypellacytosporin B | *Eutypella* sp. D-1 | Rice medium | Antitumor activity | (Zhang et al. 2019) |
| **146** | eutypellacytosporin C | *Eutypella* sp. D-1 | Rice medium | Antitumor activity | (Zhang et al. 2019) |
| **147** | eutypellacytosporin D | *Eutypella* sp. D-1 | Rice medium | Antitumor activity | (Zhang et al. 2019) |
| **148** | eutyscoparol J | *Eutypella scoparia* SCBG-8 | Rice medium | Antitumor activity | (Zhang et al. 2021b) |
| **149** | pestaloquinol A | *Eutypella scoparia* SCBG-8 | Rice medium | Antitumor activity | (Zhang et al. 2021b) |
| **150** | scoparasin A | *Eutypella scoparia* PSU-D44;  *Eutypella* scoparia PSU-H267;  *Eutypella scoparia* 1-15;  *Eutypella scoparia* SCBG-8 | Culture broth;  solid medium;  rice medium | Antitumor activity;  cytotoxic activities against Vero cells | (Pongcharoen et al. 2006; Kongprapan et al. 2015; Qi et al. 2015; Zhang et al. 2021b) |
| **151** | scoparasin B | *Eutypella scoparia* PSU-D44;  *Eutypella* sp. D-1;  *Eutypella scoparia* SCBG-8 | Culture broth;  rice medium | Antitumor activity;  antibacterial activity | (Pongcharoen et al. 2006; Liu et al. 2014; Zhou et al. 2017; Zhang et al. 2021b) |
| **152** | phenochalasin B | *Eutypella scoparia* FS26;  *Eutypella* scoparia PSU-H267;  *Eutypella scoparia* 1-15; | Culture broth;  solid medium | Antitumor activity;  cytotoxic activities against Vero cells | (Sun et al. 2011b; Kongprapan et al. 2015; Qi et al. 2015) |
| **153** | [12]-cytochalasin | *Eutypella scoparia* FS26;  *Eutypella* scoparia PSU-H267 | Fermentation broth | Antitumor activity;  cytotoxic activities against Vero cells | (Sun et al. 2013; Kongprapan et al. 2015) |
| **154** | cytochalasins Z_24_ | *Eutypella* sp. D-1;  *Eutypella scoparia* SCBG-8 | Fermentation broth;  Rice medium | Antitumor activity | (Liu et al. 2014; Zhou et al. 2017; Zhang et al. 2021b) |
| **155** | cytochalasins Z_25_ | *Eutypella* sp. D-1 | Fermentation broth |  | (Liu et al. 2014; Zhou et al. 2017) |
| **156** | cytochalasins Z_26_ | *Eutypella* sp. D-1 | Fermentation broth |  | (Liu et al. 2014; Zhou et al. 2017) |
| **157** | scoparasin C | *Eutypella* scoparia PSU-H267 | Culture broth | Cytotoxic activities against Vero cells | (Kongprapan et al. 2015) |
| **158** | scoparasin C | *Eutypella scoparia* 1-15 | Solid medium |  | (Qi et al. 2015) |
| **159** | scoparasin D | *Eutypella scoparia* 1-15;  *Eutypella scoparia* SCBG-8;  *Eutypella* scoparia PSU-H267 | Solid medium;  Rice medium;  culture broth | Antitumor activity;  cytotoxic activities against Vero cells | (Kongprapan et al. 2015; Qi et al. 2015; Zhang et al. 2021b) |
| **160** | eutypoid A | *Eutypa* sp. (#424) | Culture broth |  | (Lin et al. 2002) |
| **161** | eutypellin A | *Eutypella* sp. BCC 13199 | Fermentation broth | Antitumor activity; cytotoxic activities against Vero cells | (Isaka et al. 2009) |
| **162** | eutypellin B | *Eutypella* sp. BCC 13199 | Fermentation broth |  | (Isaka et al. 2009) |
| **163** | butyrolactone I | *Eutypella* sp.ZZ2 | Solid medium |  | (Liao et al. 2017) |
| **164** | butyrolactone II | *Eutypella* sp.ZZ2 | Solid medium |  | (Liao et al. 2017) |
| **165** | butyrolactone III | *Eutypella* sp.ZZ2 | Solid medium |  | (Liao et al. 2017) |
| **166** | butyrolactone V | *Eutypella* sp.ZZ2 | Solid medium |  | (Liao et al. 2017) |
| **167** | cytosporin D | *Eutypella* *scoparia* ICB-OBX;  *Eutypella scoparia* PSU-H267;  *Eutypella* sp.ZZ2;  *Eutypella* sp. D-1;  *Eutypella scoparia* HBU-91 | Culture broth; solid medium; rice medium | Anti-inflammatory activity; antiviral activity | (Ciavatta et al. 2008; Kongprapan et al. 2015; Liao et al. 2017; Zhang et al. 2019; Zhang et al. 2022; Yu et al. 2023) |
| **168** | cytosporin E | *Eutypella* *scoparia* ICB-OBX;  *Eutypella* sp.ZZ2;  *Eutypella* sp. D-1 | Culture broth; solid medium; rice medium |  | (Ciavatta et al. 2008; Liao et al. 2017; Yu et al. 2023) |
| **169** | 7,8-dihydroxy-3,5,7-trimethyl-8,8a-dihydro-1*H*-isochromen-6(7*H*)-one | *Eutypella scoparia* FS26 | Fermentation broth |  | (Sun et al. 2013) |
| **170** | 6-(hydroxymethyl)-2,2-dimethyl-3,4-dihydro-2*H*-chromene-3,4-diol | *Eutypella scoparia* FS26 | Fermentation broth; |  | (Sun et al. 2013) |
| **171** | (*R*)-3,4-dihydro-4,8-dihydroxy-6-methoxy-4,5-dimethyl-3-methyleneisochromen-1-one | *Eutypella scoparia* PSU-D44;  *Eutypella scoparia* PSU-H267;  *Eutypella scoparia* HBU-91 | Culture broth; rice medium | Anti-inflammatory activity | (Pongcharoen et al. 2006; Kongprapan et al. 2015; Zhang et al. 2022) |
| **172** | cytosporin L | *Eutypella* sp.ZZ2;  *Eutypella* sp. D-1 | Solid medium; rice medium | Antibacterial activity;  anti-inflammatory activity;  antiviral activity | (Liao et al. 2017; Yu et al. 2023) |
| **173** | eutyketide A | *Eutypella scoparia* HBU-91 | Rice medium |  | (Zhang et al. 2022) |
| **174** | eutyketide B | *Eutypella scoparia* HBU-91 | Rice medium |  | (Zhang et al. 2022) |
| **175** | cytosporin X | *Eutypella scoparia* HBU-91;  *Eutypella* sp. D-1 | Rice medium;  solid medium | Anti-inflammatory activity | (Zhang et al. 2022; Yu et al. 2023) |
| **176** | banksialactone A | *Eutypella scoparia* HBU-91 | Rice medium | Anti-inflammatory activity | (Zhang et al. 2022) |
| **177** | 4,8-dihydroxy-3-(hydroxymethyl)-6-methoxy-4,5-dimethylisochroman-1-one | *Eutypella scoparia* HBU-91 | Rice medium |  | (Zhang et al. 2022) |
| **178** | eutypelleudesmane A | *Eutypella* sp. D-1 | Rice medium; solid medium |  | (Yu et al. 2023) |
| **179** | cytosporin Y | *Eutypella* sp. D-1 | Rice medium; solid medium | Anti-inflammatory activity | (Yu et al. 2023) |
| **180** | cytosporin Z | *Eutypella* sp. D-1 | Rice medium; solid medium |  | (Yu et al. 2023) |
| **181** | cytosporin Y_1_ | *Eutypella* sp. D-1 | Rice medium; solid medium |  | (Yu et al. 2023) |
| **182** | cytosporin Y_2_ | *Eutypella* sp. D-1 | Rice medium; solid medium |  | (Yu et al. 2023) |
| **183** | cytosporin Y_3_ | *Eutypella* sp. D-1 | Rice medium; solid medium | Anti-inflammatory activity | (Yu et al. 2023) |
| **184** | cytosporin E_1_ | *Eutypella* sp. D-1 | Rice medium; solid medium |  | (Yu et al. 2023) |
| **185** | cytosporin F | *Eutypella* sp. D-1 | Rice medium; solid medium |  | (Yu et al. 2023) |
| **186** | eutyscoparol A | *Eutypella* *scoparia* SCBG-8 | Rice medium |  | (Zhang et al. 2020) |
| **187** | eutyscoparol B | *Eutypella scoparia* SCBG-8 | Rice medium |  | (Zhang et al. 2020) |
| **188** | eutyscoparol C | *Eutypella scoparia* SCBG-8 | Rice medium |  | (Zhang et al. 2020) |
| **189** | eutyscoparol D | *Eutypella scoparia* SCBG-8 | Rice medium |  | (Zhang et al. 2020) |
| **190** | eutyscoparol E | *Eutypella scoparia* SCBG-8 | Rice medium |  | (Zhang et al. 2020) |
| **191** | eutyscoparol F | *Eutypella scoparia* SCBG-8 | Rice medium |  | (Zhang et al. 2020) |
| **192** | eutyscoparol G | *Eutypella scoparia* SCBG-8 | Rice medium |  | (Zhang et al. 2020) |
| **193** | eutyscoparol H | *Eutypella scoparia* SCBG-8 | Rice medium | Antibacterial activity; anti-MRSA activity | (Zhang et al. 2020) |
| **194** | eutyscoparol I | *Eutypella scoparia* SCBG-8 | Rice medium | Antibacterial activity; anti-MRSA activity | (Zhang et al. 2020) |
| **195** | tetrahydroauroglaucin | *Eutypella scoparia* SCBG-8 | Rice medium | Antibacterial activity;  anti-MRSA activity | (Zhang et al. 2021b) |
| **196** | flavoglaucin | *Eutypella scoparia* SCBG-8 | Rice medium | Antibacterial activity;  anti-MRSA activity | (Zhang et al. 2021b) |
| **197** | pyochelin | *Eutypa lata* | Iron-free culture broth |  | (Perez-Gonzalez et al. 2022) |
| **198** | 3,4-dihydroxybenzoic acid | *Eutypa lata* | Iron-free culture broth |  | (Perez-Gonzalez et al. 2022) |
| **199** | 3,4,5-trimethoxycinnamic acid | *Eutypa lata* | Iron-free culture broth |  | (Perez-Gonzalez et al. 2022) |
| **200** | polygonolide | *Eutypa lata* | Iron-free culture broth |  | (Perez-Gonzalez et al. 2022) |
| **201** | 3,4’,5-Biphenyltriol | *Eutypa lata* | Iron-free culture broth |  | (Perez-Gonzalez et al. 2022) |
| **202** | 8-methoxynaphthalen-1-ol | *Diatrype palmicola* MFLUCC 17-0313 | Culture broth | Antifungal activity | (Tanapichatsakul et al. 2020) |
| **203** | lunalide A | *Diatrype* sp. |  |  | (Williams et al. 2008) |
| **204** | lunalide B | *Diatrype* sp. |  |  | (Williams et al. 2008) |
| **205** | *cyclo*-(L-Pro-L-Leu) | *Eutypella* *scoparia* ICB-OBX | Culture broth |  | (Ciavatta et al. 2008) |
| **206** | *cyclo*-(L-Pro-L-Phe) | *Eutypella* *scoparia* ICB-OBX | Culture broth |  | (Ciavatta et al. 2008) |
| **207** | eutypellazine A | *Eutypella* sp. MCCC 3A00281 | Rice medium | Anti-HIV activity | (Niu et al. 2017a) |
| **208** | eutypellazine B | *Eutypella* sp. MCCC 3A00281 | Rice medium | Anti-HIV activity | (Niu et al. 2017a) |
| **209** | eutypellazine C | *Eutypella* sp. MCCC 3A00281 | Rice medium | Anti-HIV activity | (Niu et al. 2017a) |
| **210** | eutypellazine D | *Eutypella* sp. MCCC 3A00281 | Rice medium | Anti-HIV activity | (Niu et al. 2017a) |
| **211** | eutypellazine E | *Eutypella* sp. MCCC 3A00281 | Rice medium | Anti-HIV activity | (Niu et al. 2017a) |
| **212** | eutypellazine F | *Eutypella* sp. MCCC 3A00281 | Rice medium | Anti-HIV activity | (Niu et al. 2017a) |
| **213** | eutypellazine G | *Eutypella* sp. MCCC 3A00281 | Rice medium | Anti-HIV activity | (Niu et al. 2017a) |
| **214** | eutypellazine H | *Eutypella* sp. MCCC 3A00281 | Rice medium | Anti-HIV activity | (Niu et al. 2017a) |
| **215** | eutypellazine I | *Eutypella* sp. MCCC 3A00281 | Rice medium | Anti-HIV activity | (Niu et al. 2017a) |
| **216** | eutypellazine J | *Eutypella* sp. MCCC 3A00281 | Rice medium | Anti-HIV activity | (Niu et al. 2017a) |
| **217** | eutypellazine K | *Eutypella* sp. MCCC 3A00281 | Rice medium | Anti-HIV activity | (Niu et al. 2017a) |
| **218** | eutypellazine L | *Eutypella* sp. MCCC 3A00281 | Rice medium | Anti-HIV activity | (Niu et al. 2017a) |
| **219** | eutypellazine M | *Eutypella* sp. MCCC 3A00281 | Rice medium |  | (Niu et al. 2017a) |
| **220** | eutypellazine N | *Eutypella* sp. MCCC 3A00281 | Rice medium |  | (Niu et al. 2017b) |
| **221** | eutypellazine O | *Eutypella* sp. MCCC 3A00281 | Rice medium |  | (Niu et al. 2017b) |
| **222** | eutypellazine P | *Eutypella* sp. MCCC 3A00281 | Rice medium | Antimicrobial activity | (Niu et al. 2017b) |
| **223** | eutypellazine Q | *Eutypella* sp. MCCC 3A00281 | Rice medium | Antimicrobial activity | (Niu et al. 2017b) |
| **224** | eutypellazine R | *Eutypella* sp. MCCC 3A00281 | Rice medium | Antimicrobial activity | (Niu et al. 2017b) |
| **225** | eutypellazine S | *Eutypella* sp. MCCC 3A00281 | Rice medium |  | (Niu et al. 2017b) |
| **226** |  | *Eutypella* sp. MCCC 3A00281 | Rice medium |  | (Niu et al. 2017b) |
| **227** |  | *Eutypella* sp. MCCC 3A00281 | Rice medium |  | (Niu et al. 2017b) |
| **228** |  | *Eutypella* sp. MCCC 3A00281 | Rice medium |  | (Niu et al. 2017b) |
| **229** | epicoccin A | *Eutypella* sp. MCCC 3A00281 | Rice medium | Anti-HIV activity | (Niu et al. 2017a) |
| **230** | epicoccin I | *Eutypella* sp. MCCC 3A00281 | Rice medium |  | (Niu et al. 2017a) |
| **231** | 4-Methoxy-3-(3-methylbut-3-en-1-ynyl)benzyl Alcohol | *Eutypa lata* (Pers: F.) TUL. | Culture medium |  | (Renaud et al. 1989b) |
| **232** | eutypinol | *Eutypa lata* (Pers: F.) TUL.;  *Eutypa lata* strain E125 | Culture medium;  PDB culture medium |  | (Renaud et al. 1989b; Molyneux et al. 2002) |
| **233** | 4-Methoxy-3-(3-methylbut-3-en-1-ynyl)benzaldehyde | *Eutypa lata* (Pers: F.) TUL. | Culture medium |  | (Renaud et al. 1989b) |
| **234** | eutypine | *Eutypa lata* (Pers: F.) TUL.;  *Eutypa lata* strain E125 | Culture medium;  PDB culture medium |  | (Renaud et al. 1989b; Molyneux et al. 2002) |
| **235** | eutypinic acid | *Eutypa lata* (Pers: F.) TUL. | Culture medium |  | (Renaud et al. 1989b) |
| **236** | 4-Hydroxy-3-(3,4-dihydroxy-3-methylbut-1-ynyl)benzyl Alcohol | *Eutypa lata* (Pers: F.) TUL. | Culture medium |  | (Renaud et al. 1989b) |
| **237** | 4-Hydroxy-3-(3,4-dihydroxy-3-methylbut-1-ynyl)benzaldehyde | *Eutypa lata* (Pers: F.) TUL. | Culture medium |  | (Renaud et al. 1989b) |
| **238** | 4-Hydroxy-3-(3,4-dihydroxy-3-methylbut-1-ynyl)benzoic Acid | *Eutypa lata* (Pers: F.) TUL. | Culture medium |  | (Renaud et al. 1989b) |
| **239** | 5-formyl-2-(methylvinyl)[1]benzofuran | *Eutypa lata* (Pers: F.) TUL. | Culture medium |  | (Renaud et al. 1989b) |
| **240** | eulatinol | *Eutypa lata* strain E120 | MYB medium |  | (Molyneux et al. 2002) |
| **241** | siccayne | *Eutypa lata* strain E120 | MYB medium |  | (Molyneux et al. 2002) |
| **242** | eulatachromene | *Eutypa lata* strain E125 | PDB culture medium |  | (Molyneux et al. 2002) |
| **243** | 5-(3-methylbuta-1,3-dienylidene)-2,3-epoxycyclohexane**-**1,4-diol | *Eutypa lata* (Pers: F.) TUL. | Culture broth |  | (Renaud et al. 1989a; Defrancq et al. 1992) |
| **244** | eutypoxide B | *Eutypa lata* (Pers: F.) TUL. | Culture broth |  | (Defrancq et al. 1992) |
| **245** | 6-hydroxy-2,2-dimethyl-5,6,7,8-tetrahydro-7,8-epoxychroman-4-one | *Eutypa lata* (Pers: F.) TUL. | Culture broth |  | (Renaud et al. 1989a; Defrancq et al. 1992) |
| **246** | 8-hydroxy-2,2-dimethyl-5,6,7,8-tetrahydro-6,7-epoxychroman-4-one | *Eutypa lata* (Pers: F.) TUL. | Culture broth |  | (Renaud et al. 1989a; Defrancq et al. 1992) |
| **247** | terrein | *Eutypa lata* | Iron-free culture broth |  | (Perez-Gonzalez et al. 2022) |
| **248** | cladoacetal C | *Eutypella* sp. D-1 | Rice medium |  | (Yu et al. 2020) |
| **249** | benzophomopsin A | *Eutypella* sp. D-1 | Rice medium |  | (Yu et al. 2020) |
| **250** | pestalospirane B | *Eutypella* sp. D-1 | Rice medium | Antitumor activity | (Yu et al. 2020) |
| **251** | uracil | *Eutypella scoparia* FS26 | Fermention broth |  | (Sun et al. 2011b) |
| **252** | *α*-Linolenic acid | *Eutypella* sp. D-1 | Fermention broth |  | (Liu 2016) |
| **253** | (*R*)-1-(2,3-dihydro-1*H-*pyrrolizin-5-yl)-2,3-dihydroxypropan-1-one | *Eutypella* sp. D-1 | Fermention broth |  | (Tan et al. 2017) |
| **254** | vincristine | *Eutypella* spp - CrP14 | Fermention broth | Antitumor activity | (Kuriakose et al. 2016) |

# Table S2. The predicted terpene synthase from the genome of *Eutypella* sp. D-1.

| **Name** | **Contig** | **Location** | **Amino acid sequence** | **GenBank Accession No.** |
| --- | --- | --- | --- | --- |
| Eut-TS01 | JAJTTP010000001.1 | 1,237,696–1,239,364 | MSNKHFGAHDFVQTLRGQKLKIPNLDAVVEGWPRGCSPHLKYLRDVQHKDLFKLLGPGKAFDICESTDCPYFAANWWPNASWDALRYAAQLVTFLYLWDDDTDNPELTDLVSDLDASNNFRDASNRHFERYLADNPPPGNPADPPVPTTLRSFNPIGEAAAFRMTRGQRLRLLAELHRYMEACATEQHSELSGRAPTVDEYLPCRMGTSAAGFVVANLEYMLGVDLGDTIRYDPDLVEVFEQTICLIAIMNDMFSLRRELRYPFYNNIVAVLYAEHRNLQTAVDETYKIIQRSANTLEAAAKRALNRYPERREDITTWINGAKTMVTGNMAWSMHIKRYSLGVKDLDGNAK | PP432627 |
| Eut-TS02 | JAJTTP010000001.1 | 3,983,592–3,986,341 | MTTKPSEAPWADEPFNLIATPSKRIKDDHSYVRVASDMAHAHNVIIRGLNSIVQQAPYVPSSSDQGYNARDVKDLLFYAQSWVKMVNHHHWVEESFIFPEMEKVSGRPGLMDDPRHQHELFHPGMERLLGYCSATTPEEYRWEGGMKDIIDSFSKELTDHLLGVGQAWDKAETIAKQAGNIAMLYDVFPCVLGCADKTYEGGHPFPPLPWFMPYLVKYWFAAGNNGWRFNPCDWWERRLKLELRGIFQKAHPKPHIIYVSDNSSSLGFSHEKKKQLEVVDFGLFIALWWPHVQFGKLKTLTYLVIWLFTWDDEIDEPTGAYASDFDGAQTYREHTLRFVGKCLGVATVETELRPLNRITQSFDIIGASLRSSYDFEQRQRFYDTIAHFMEASEAEQRFRLEGQVPTLEQYWEFRLGTSAVYISSAAAEFSMSAKLPSEVMSCKDMGIIWDETNFIISITNDLASLKKEMRLDCMDSIVPLTFAMTTDIQAAISKSLEALRRSKKRFDEATRALLSAQVGDEENYQKVKQFIDVQRSNCVGNLLWSLETQRYNLLEVITQDSVMDAWLAFVEGIQNDCLLS | PP432628 |
| Eut-TS03 | JAJTTP010000001.1 | 4,164,543–4,165,713 | MIETIPTVAAPAKTADAAFKSFKTMPFTEHFNKLQIPASALVVRTHPLTDKVADAVNQFYLDTWPFPGEKERKKFIGADFPRFTCYYFPNTRDDRLRLVCELVTHLFLVDDIIENMSLKEGRAFNDKLIALARGEVLPDRTSPPEWIMYDVWQGFRKCDKELADATLQPCFDFMNSQTDPRRLTKMNLEQYLDYRLDDVGKEFLSALICWTQGLHLTKEDWALVDPIDKNSARHISVINDIWSYDKELKATALNQEGSTMLNAVDILADEAFMPIESAKRVLYKLCREWEIVHETLVDHVLSKRDTPTLRAYIKETEYQMSGNEIWSRLTLRYSQIH | PP432629 |
| Eut-TS04 | JAJTTP010000001.1 | 5,371,779–5,372,768 | MQPMLAFHIPKVLFRGKRSRSGFATHISAARRIPWTHFFPKAANFIVTASLSFTNVTALAGCEVPRMARTTGVDGSTWSQYMREKDGVGEAYGWFTFPKALYPDLSEFMEVVPDIGSYVCIVNDILSFYKEEKDGEKHNYMHNRSTYERTDVYTTLKAAIKEAVGIQHRMQRILRGKESHMRACNDHCMGLSELGLGEKYTDQFGNLTSLRQDI | PP432630 |
| Eut-TS05 | JAJTTP010000011.1 | 446,115–447,431 | MAPSIDQVQPITIDPVDQRKNVVKRALNQKVLVPNILSLMPAWPSNLQPDIDEVNKDIDEWLKTVDVAEEKKIKHRARGNYTLLTAVYYPDCKREKMLALTQFLYWIFFYDDGGDLTEDKHAAMEVTMATKKCVDDCLGPNPSYSPPPGSKGVVVMFYQILRDLREGMGPTSTERLRNELHDYLDGVVNQQEVRLEDGLPNPWDHFRMRSDDVGVIPSITQNEYAMDFELPEWVRRHEAMEEIVQECTKLTTLLNEVLSLQKEFRVNQLENLVLLFMNEYTMSLHSAIEKVLDLIREHYAICVAAEARLPWSDHDEKFNADLREYVAGCHRLATGTTYWR | PP432631 |
| Eut-TS06 | JAJTTP010000012.1 | 11,495–12,496 | MYKKAVGYGIPLDNPLSAKGFRLGFSEGALAYPNHPVEIQAYIGLFTWIVVQIDDVIGDIKEEADDFQRRFFSGERQAHCLLHGLAEMLREAYDHWDPVLANILVTSGLNFLTSNLLETREGFQKMPVTKAGRSFPYYYRDLAGITEAYAIFGYPTAVYPDIENFLEAIPDMALFINIFNDVVSFYKEELGNDTRNYIHNRAMCEQKTALKVVKEVKGEVVDCVNRIRSVLKGRGIYAESWEMHMRGHIAMHTTNPRYRLKELGLGEENPFPEALYKEMEALTQGSIND | PP432632 |
| Eut-TS07 | JAJTTP010000014.1 | 218,900–220,008 | MTDSTKTVTIQGYTLPEEPMSHCGPPPPAPAASQVPGDIRPTIYTAGMHRKTKEVVEQTNDFFLQNWPFKTEKHLKRFVEEGYACPMSLDERMHWGCKLLTVGFLIDDLLDSMSVEEGVAYNTKVIECARGTMLPDRSVPGQWIMYDLFENMRAVDKPLADELLVHTVDFLFAQVDGSRTKPMDLKEYFVYRDADLGKGLMTAIMRFCAGLYMTPEEQDLVKPLEDIAMKHITFVNDVCSFEKELLSAANGAELGAICSSVPIIMDLYGVGEYEAKRIMWQTVRAWEVRHFELVDEILRKNSSPALRTYCKGIEYQTAGNERWSLLTPRYNKTGALAFTEGR | PP432633 |
| Eut-TS08 | JAJTTP010000023.1 | 345,555–348,045 | MVETRSTAKSMANGYEKPNGNGNGKKARKRTSADDLNGETKRPRLEDKTDRTRWRMLDDKGRHTWHYLEDDEAVKKWPQSVADKYYLGLDTEQPDLPKPQTPLDAVRNGLTFFEKLQLPSGQWGCEYGGPMFLLPGIVFTWYATKTPIPWYVATEIKNYLFARANPEDGGWGLHIEGESTVFGTALNYSVLRIVGVDPEHPVMVKARGTLHKLGGAVYGPHWAKFWLSVLGVCRWEIVNPVPPELWLLPDWVPFAPWRWWIHMRQVFMPMCYIWDKKFTCEETDVIRGLRQELFVEPWEEIDWAGNRNTICPRDNYHPKSWLLNTANWFLTNIWNPYLRTKSLSDRACDWISKLVDMEDANTDFADLAPVNAPMNTIVCYIRDGPGSYTVRRHIERLEDALWMNKEGFLCNGTNGVQSWDTAFLIQSVVDAGLEQDPRWRPMLTKALEFLDDQQIREDCAEQGVCYRQRRKGAWAFSTRVQGYAVSDCISEALKSVILLQKTPGYPQLLEDQRFFDAVDTLLTYQNATGACSSYEPTRGSAHMEMLNAAEVFGKIMIEYDYVECTTAVITALKLFREHWPKYRNQEIEAFIARSLKWIKTYQRPDGSFYGNWAICFTYATMFALESFHSIGEHYENSSASKRACDFLLSKQREDGGWSESYKADYPDIEPIKKGIKLIMDRQQPNGEWLQEAIEGVFNKSCMISYPNYKFTFTMKALGMFASKYPDETVV | PP432634 |
| Eut-TS09 | JAJTTP010000005.1 | 306,960–308,183 | MDSIVAAANPGSDTDGPFTTNPPLSPQSPNAIPPRTSSTGHAVPHLPSKSALRPLPESNWLGQSAQRHRKSSSATAVPSIAMLNNNPPPDPSRFATEELNITTQKQWTADKEKVVLGPYDYLYSVPGKDFRTLMVNTFNAWLEVPKESLDVITKVVGMLHTSSLLIDDVEDNSILRRAMPVTHSIFGTAQTINSANYVYICALQELKKLNNPKAADIYTEELINLHRGQGMDLFWRDTLTCPTEEDYLEMVGNKTSGLFRLAIRLMQAESRSSIDCVPLVHILGLIFQIEDDYKNLSDDKYTENKGFAEDLTEGKFSFPIIHSIRSDPTNLQLLNILRQKTNNVEVKHYAIKYMESTGSFDYTRRVIEVLIERARKIVDDVDAGRGRGHGVHKILDKLVMSQQATAS | PP432635 |
| Eut-TS10 | JAJTTP010000007.1 | 1,693,340–1,695,277 | MGWDYLLVHVKFTIPLAGLLTFILRPLLSRTDLYKTSALIVIAFTAALPWDSYLIHQRIWTYPEDAILGLTLCAVPVEELFFFVVQTYITSMFYILVNKTSFHPQYFSNKSDSSPLAKKVRRYGQYTLTVFIAIGVYLVRKGQEGTYLGLILAWACPFALLTWTFSGLFIVRLPSARVVLPILLPTLYFWLVDELALGRGTWGIESGTKLDIRLFGDLDIEEAVFFLATNCLIVFGLIAFDRGTAVVATFPHLFPEVPETPSLSFVLRGVFTSAEKYDVARVRGLRDAVSRLRKKSRSFYLASSAFPGRLRIDLIILYSFCRVADDLVDEARSQEAALDWIDKLTKYLDLAYISDSEKAAATTHSLDSFVDGNFPESARTALKLLPTKCLPREPFYELLEGFRMDLSFTQSDSPPSGGGVIHFPIEDEQALELYAHRVASTVGELCLWLVFYHCQTRSLPEDTKTKLIGAARTMGHALQYVNIARDIRVDAAMGRVYLPTSWLRQEDGGLTPADIVRDPGQPAAERLRQRLLDLAFAEYARARPIMSALPGEVRAPLVVAVESYMEIGRVLRERAGIPSGKDPRRATVPRIRRLWVAWKNLSSV | PP432636 |

# Table S3. The predicted *scop* BGC in JAJTTP010000033.1 in *Eutypella* sp. D-1.

| **Name** | **Location of gene** | **Amino acid sequence** | **GenBank Accession No.** |
| --- | --- | --- | --- |
| *scopA* | 97,900–98,689 | MAVIEIQVISDIVCAWCYVAKRNLDQAISLYRKTYPGGKSDTFAITWTPYYLNYNPHSCSVEKHTLVDVRLPDTTPEQREAMGKKMNAAGRAVGINFNWGGKIGPDTRDAHRLIHLAQTRRSGDHGNVDPDGEALDAVVEGLFEAYHVLAQDVSEKEVLRNIALRAGIQAAEVDRVLDSDEGADIVDKQAEESKKITNAGVPMLIIQGSHRVDSTPDPMDLMLLFVKVREEQTEGQPA | WWS34683 |
| *scopB* | 100,573–102,463 | MKNGLPDRQTVICEGSDHLLRVEPNAPLATLGPGDILVRNKAVAINPCDYKMHERFPCTGAIDGCDFAGVVVAVGSQVSESGSFQVGDRVCGAVHGSNPLRPECGSFAEYVVSESEFTLKIHPDMSFEEAAALGGTGLATLGMALFHESALGLPGTPDAPAAKPRTVLVYVCSSKNSSLARSYGAEEVFDYTDPDCAKEIKTHTRNSLSYVLDPFTDAKSIALCYGAMGRAGGRYSCLEMYPDYLLDNRLIKVGFVMGPALLGHRLALDHGYERDADPAMRAFGVKWYNSRHEYYPPGFALDHLVYHHTYNLLAYIATAAVLYRIIYWLLYPGGHRPVTKKSLQHIPELKFEENDTFQRYLQETRSLIFRGYEKYLKHGTPFQMRNPVGELGPQLMLPMKYMDEVKNAPTSLFSFKAFSEKMFFLNYSDAPLQSEAATHVIKVDLNRNLGARFQ | WWS34684 |
| *scopC* | 102,537–104,149 | MFDRLAASAKQSAWLEPWPADWIEKASLQFNDPQQAPLGPDGGWGGGQCESSNLTAFEMAKGRQAVDFTTKAVEDLELPKIRPLNSSGGEQWEFDGVSEDGMQSFIFGFYRDPNYAILGTGNFRLSIEFAFADRTRFYEVYYPQRSVVETCGSVGTRGTWFDEADGYSFMFQVNADMSEAIITMDSDTVKGRAVIRSRSKPVTADGHVWPSENASTAPVPYWHWSEPIPGGDAELDVNIKGKQITWSGMGGHERFWSAFSSVKAKLGPYVLTYFSFTSNMVKDLTHQSVVLFKDGAPVFRSTLGSPSETEPYALVSKTYGGAVTGTLKDKVTGFQLELVSPASMQHYTFFVEHANLGFEYILGEGVGGSGFSSVSRGGHVGLSQYEGAALTEALTFPKNSPLFRSNYVE | WWS34685 |
| *scopD* | 104,868–106,276 | MHKLFPNSHFFDFETIRILGTAAYGGADAAEVLEAVGQIKSEDPITWETAWRAQAERAEALAEEACQRGDRDAARRNFLKAASYTRASGYMYTSSPGESGELVQDSRALPVSEKVGKLFRQAVPLMEGQVHSLSIPYEEYALPGYLYLPQEGRRIPGRSKIPILLVCGGADSCQEELYFMNPAAGPGLGYAVVTFDGPGQGLMLRKYGLEMRPDWETVTGSVINHLASFSAAHPELDLDMECIAVSGASMGGYYALRAASDPRVRACVAIDPFYDMWDFGTAHVNPLFIKAWTGGWISSGLIDRMMGLLSKLSFQLKWEISVSGTFFGISSPSEILLNMKKYTFNNKNPLEEKTGDSFLSRISCPVRCYEALRNVPGVNKEVWIPSTEGQGSLQAKMGAMALCNQKTYQFLDKAFGVVREPLNQGSLL | WWS34686 |
| *scopE* | 107,613–108,799 | MADLTRSLPARVFSRQQDCRMAFDTVIGYCPEWKEFDLLLTTFELVAQINACSFVGRELGCNRKWVRAVMMSPIVIHVAVTMMNELPNMLRPLLAPLLFLPALKNQWDMKRLLTPYLKDDIKTFEGYADKKELLKPNPEGKIPFTAMLLARYKAAEASIKQLVEDYILVSFDSTPSTTSALYHIICELAKHPEAVDILRQELDEVMVDGKLPSTHLQELKRMDSFLRESFRMHPVSLFTLQRYTEKPVKLSVGPTIPAGAIIGVDAQAINRSPELWEEPDKFDMNRFYNLRQQPGNENRYHFLSTGPDSPGWGDGTQACPGRFFATSTIKIAFAHILLNYDIELKDSTRDIKITPLANGTWKPDDTVVVRFKSRT | WWS34687 |
| *scopF* | 109,892–123,432 | MTTKDPIAIVGTGCRFPGQCDNPSKLWELLQEPRDLLKEIPEDRFIYESLEAAGLSPNQLRGTDTAVYVGVMSADYTDMISRDINTFPTYFATGTARSILSNRLSHFFDWHGPSMTIDTACSSSLIAVHQAVQSLRAGESSVAVVAGSNLILGPEQYIAESKLQMLSPTGRSRMWDADADGYARGEGVAAVVLKRLSQALADGDHVECIIRETGVNQDGKTPGITMPSASAQAALIRSTYARAGLDLSQRSDRPQYFEAHGTGTPAGDPIEAEAISNAFFGPGSNFNPRKGEDPLYVGSIKTVIGHTEGTAGLAAIIKASLALQAGKVPPNRLLNQLNPKVAPFYGNLKILSAAEAWPRTTGDAVRRASVNSFGFGGANAHAILESFDSHSNKALPSRQTQSDTCFTPFVFSASSDSALGSNLQSYRDYIVEATTHSDIPYTLRDLSWTLSSRRTALEYRTVLPAVSGFEDLITKLDEIIESQDEFHQSTRSPPAGTKLRILGIFTGQGAQWARMGAELIEKSPEASRIIEKLDRSLASLPLDDRPPWSLRDQITASPEQSQINTASISQPICTAIQVMLVDLLTAAGVEFSTVVGHSSGEIGAAYAARYLTAEDAIRVAYYRGLHLRGTPEKKGAMMAVGTSFEDLSELCELPAFEGRVCVAASNSQASVTLSGDADAVEEVKVVLDEEKKFARLLKVDRAYHSHHMAPYADPYIRSLQGCNIKPKAGSHCRWVSSVFVEDIANIPQNESLGGKYWTSNLVKPVMFAEALSKALDSKDDSYDLVMEVGPHPALKGPASQTIQNSLEGQSIPYTGTLSRGKDSIQAFSTALGYVWESLGEGAVNFAAFDAFVTGNQSPKPKLCKGLPTYQWDHNRVFWHESRASKAFRTSKDGHNELLGKQFFDGAPDQLRWRNVLKRREIDWLEGHQVQGQVVFPCAGYVSACIEAAMKLPGTNKQVDQGQGQQLVVQTVELEDFIVGQAVVFGDDQDSGIETLVTLTDINWDHSETQDGSVAVAARFAFYSSPNNDASEMTSHASCRVRVRLAQDDAENAAKNSALPGKSQVDDVGMAEVESDRFYDALGKLGFGYSGPFKALKELRRKLGIASGLIHNSSPLAPSPPLIVQPATLDAAIQSIMLAYCYPGDSMLRSIYLPTGIKRLIISPQRCLTFTGADADVHFDSNASINTSRGLSGDVSIYALDGRSKAIQLEGLQTKPLSNPTESSDLNIFTELVWEVEEPDSEAVIATTPAPELNADLLSSLERVTYYYLRSLETKFPRKERGNLNLEWYQERLFAYVDHCLAKVVRGANPYAKPEWSHDTEATILEIFNRYPDNIDLRLMRAVGENLPSVIRDKGTMLEYMIHDNMLNDFYVVAHGMPRYTKYLASMASQIGHRYPHMSVLEIGAGTGGATKSFLKELGDSFSTYTFTDISSGFFAKAEETFASYSSRMNFKVLDIEKDIEEQGFADGSFDLIIASLVLHATRNLADTLKNVRRLLKPGGYLLLLEITENEQMRFGLIFGGLPGWWLGYDDGRALSPCVGIDEWKHLLKRTGFSGIETVIPHHETLPVPLSIIASQAVDSRIDFLKKPLLSPTPALFSPSAPVIHRLTLIGGGGSKSAQLAEDISNILSQDDRHCGETRFIASLEDIRPDQDLPIGGTTLSLVDADEAVFKSMTAEKLRGFQEIFKQSANVLWITQGSRFGEPFARMVVGFGRTLVLEMLHLRLQFLDLSPDETAGGIATTSNAIAIVESVIRFEAARAWVGDSGALDGQQPLLYSTEPELYLEPETKRLYIPRFKLNKSQNDRYNSGRRTITQQVDGRETALQLVPRTDGTCYLLEDAQSPNATAHPSGVSSQTVEIDILYSVSQAIEVLKDSFLFPVLGRDRVTGEKILALSPKQSSRISVPKTFVLPGVAIPEEQEIAVETLQKLYTELLAQSMASDVLADTQLVLLQPDPALAQIVSRISTDKGAKLTCLGLTTRERSSRDSESGLDWRYAHPMASKPEIHDLVHDLIFSAGSGMAAATWLIFNLGGPSAASFAQSLVGCLPQEITTQLRSEGNWKLSAKSLSPPPGVDVQGQTIRDLLIEAKYGMIVQDSTQEPGSRRMNVVSVGKRVDQEHAYRDDSPHVISWDSDQGALPVQVMPVDNHIRFRSDKTYWLVGLTGGLGLSLCEWMAQHGARHLVISSRSPKVDERWLKKMKWLNVHVEVLANDVCDRASVHAAYSKICQIMPPVAGVAQGAMVLHDTMFLDLDMERISKVMGPKVKGTMHLEEIFRETKLDFFIFFSSMAAVTGNPGQSAYAAANMFMSSLASQRRRRGLNASVVHIGAIFGNGYVTRELTLAQQEFLGKVGNLWLSEQDFRQLFAEAVLAGQASRGKNPELSTGLKMIDGSQESEDTITWFRNPMFQHCIRSEGQEADQLMGADGLRDRRGVPVKAQLQDAINPAEVHEIISEAFAAKLQSSLQIEEDRPLMDLRADTIGIDSLVAVDIRSWFIKELQVEIPVLKILSGATMGELIANAQELLPQTLTPNLDPNNKDKPRQKDAAKVKAKKEPVQPEKPKAPEPQQQKKQPTQASAQESKPNGIVSAGKPRQQANAFGGPSATHTQPGNAEDRREEPIKGSSSVAPSSKSIAHPDSDGSRPPSQADTLTSSFDKIAPPSVADTPAYSTSNTWSEIDDSELRSQSSNETPFTSTTKSIRETKSVASAGAQAVSDVSVTKRVPIAFAQSRFWFMEHFLQDASIASNITLCIDLEGSLDVEKFGRSVKLIGQRHEALRTRFVPVEGDDAAASKYDVMQEVLASPTLALEVRDIDNEAQADDVYKEVQGHRYKLTEGQHMRVVLLRRSAASFRLIIGYHHINMDGVSLEVILRELQVAYDSTTRLPSVQHILQYPDFAVKQRRAYESGQWNSDLAFWRKEFDGGSNPAVLEPIPLLPLTKSSWRSPLVQYSTSTAEFHIDQKLLQSIQAACGRLKVTPFHFHLAVFYTLLIRLVDVENLCIGISSANRGHEANMLQSVGLYLNLLPILFKSQPNLTFTNVLRMVRDKSLAALSHSKVPFDVIVSELDVPRSTTHNPLFQVLVNYRPGVSERRSFCGCETKATAFEQAQTAYDLVLDVIENPGGDCRVMLAGQSALYDAEHMDMLKDMYKQLLLSFARNPALRLSMASLYDANDVKHGVELGRGSFYKHTWPETIPDRIDEMVKRYSGKTALIDAGQGSKTPSSSLTYAQMADRVNTIAAAMRNSEPPVSSGSVVGVFLQPGMEWICSLLAVLRLGAIYVPLDPRTGFSRLSVIVQDCKPAAILVNNSTEQDSKGFDSVGTRINIDQIDISNHTAVSNKAEGNSVAAILYTSGSTGVPKGIVMKHETFRNNIEIMTTESYFREGLDATLQQSSYSFDMSLSQTFVTLANGGTLHVVPKELRGDPTAISSIIATQGITFTIATPAEYISWIRDGNADLGKSDWVLAQTGGEPVSKALANSFQEVGKASLKLVDCYGPTEITFCCGSRDVDYWAETSDKRHQDVDGANVAGLKTWPNYSVCIVDANTKPVPAGVPGEVLIGGAGVVAGYLHSELDARGFSRDSFASTEFLESGWSRLHRTGDLGKLSRVDGSLTLLGRIAGDTQVKLRGLRIDLKEVEAAIIDTAEGKIADAAVTVRESDTTGSEFLVAFVTTTASHGDANSDFSDVLHRLPLPPYMRPAAIVHLAKLPTNVSNKIDRNALKSLSIPQGGGNINENADTDSLNSLLDDNESRMKQLWEDVISKEVLSKYQITPESDFFHVGGNSMLLIKLRTRIQKEFGTGQILLSQLFDTSTLGGMVGLVTSHLTAPERPAGDDGNTVVEGGGEEKVIDWEEETAVSPSLVRVPASKQQFFTQPEVVVLTGGTGFLGRAILKRLVEDGVVRKIHCLAVRDPKGARDRYPSLFGSQQVVIHAGDLASPRFGLASEQQLLDIFSEAHVVIHNGADVSFMKTYASLKAVNVDATKELVRLSLPHQLSFHYISTAAVTHLTGERSFEQRSVGLYPPPTSPSDLQTKVGGYLATKWASERYLDKVSDRCELPIWIHRPSSITGDGASETDLMSNLLTYSRTTGLVPDTSSWHGWVDMISVERVAMEIADQVYEDYSWPGNVKYLFESGEQEIRLSDIKGVLERENGGRSVETVMMEDQQIGELIDSYGQAPSSRARYASA | WWS34688 |

# Table S4. A-domain sequences of PKS-NRPSs with tyrosine as substrate.

| **Name** | **A-domain location** | **Amino acid sequence** | **GenBank Accession No.**  **and reference** |
| --- | --- | --- | --- |
| TenS | 3,211–3,717 | AIKDGRNELSYAQLASRVNRTASAILGTGCSVGSRIAVLCNPSIDAIVAMLAILHIGGVYVPLDTSLPEARHQSLASNCTPSLIISHAATRERAHKLSAAISAPGHEPARELTLDDLSPPEETGYMAPLNAEPNAPAILLYTSGSTGTPKGVLLTQANFGNHIALKTDILGLQRGECVLQQSSLGFDMSLVQVFCALANGGCLVIVRQDVRRDPVELTTLMTQHKVSLTIATPSEYLAWLQYGSDALAQATSWKNLCMGGEPIPPLLKDELRRRLERKDLVVTNCYGPTETTAAISFQSVALDSEHGHELPGESELAQYAVGKALPNYSIRIRDSAGGAWLPVNHTGEIVIGGAGVALGYLDMPEETRARFLQTPGEEDGMMLYRTGDKGRLLSDGTLLCFGRITGDYQVKLRGLRIELGEVEAALLQASHGLIHTAVVSRRGDVLVAHCARSHESSRETTGGGEQQDATAILRRVSELLPQYSVPAAIALLPSLPTNANGKLDRK | A0JJU1.1  (Eley et al. 2007; Halo et al. 2008) |
| ApdA | 2,974–3,470 | PEETAIKDGKSELSYSQLTRSVEKLAAMLISQGVTAGDSVGVLLHPSIDAIACMLALLRVGCIYTPLDTRLPVARLSIIVNRSKSSLVLYHASTHDVALELGKFSKLANVEDMCESGQAQVPAIAPQSNPASFLFYTSGSTGTPKGILLSQQNFVNHLAAKTDKLNLGREVVLQQSSLGFDMSVVQTFCALGNGGTLVIAPKEARGDPIALSTIMAKERVTLTIATPSEYSLLLRFGLEQLQRPYSWRHACMGGEVVSRQLVQQFCQLDHPDLQLTNCYGPTEITAAATFQDISLQMKDQSTTDGSLVGKALPNYSVYIMDASSGSPVPIGVTGEICIGGAGVSLGYLNSLEQTDAKFVRDPFASPEDITRGWTKMYRTGDMGCLTEDGTLIFMGRMDGDNQVKLNGLRIELDEIANSILTTGNDLVSEAVVTVHSGSGSGSPLLVAHVVPLGDNVDNSRLQQLARDLPLPQYMLPSVVVSLDRLPINANGKVDRK | Q5ATG8.1  (Bergmann et al. 2007; Xu et al. 2010) |
| AsolS | 3,100–3,601 | AEEPAVTDGSATISYVQLASRVAGVSDAIIAAGFTKGTRVASLCEPSIDSVVSMLSILQAGCIYVPLDTSLPVARLVTMLAEGEPSLLIHHAATKEFVEELSAESEVSFQQLRVDEVFEGTPAFEMQMPCDLDPNATAIMLFTSGSTGKPKGIMLTQGNFSNHIALKTQALDFGREHVLQQSSLGFDMSLIQTFSALANGGLLVMASQDARRDPVELVKLQRDHRVSLTIATPSEYSAWMRYGSISLQDHAAWKNACMGGEPVTAALKKEFRRLGLNLRLTNCYGPTEITAAATFQPITLDEDYDVKDGEVDEQSEDHLRAKYAVGKALPNYSVRIVDSAGRAQSVNYTGEICIGGSGLALGYLGLPEENRTKFFVDPMMGERLYRTGDQGRLLSEGTLLCFGRIDGDSQVKLRGLRVELQEVETALLAASNGVLQDVVVSKRGESLVAHAATKPDYQHAVNDAELRNILSRLKLPQYFIPAAIVVLPSLPTNANGKLDRK | BBJ34510.1  (Haga et al. 2013) |
| DmbS | 3,192–3,690 | AIKDGRTELSYAQLASRVNHTASALVDAGCSVGSRIAVLCNPSIDAIVTMLAILHIGGVYVPLDTSLPEARHLSLASSCTPSLIISHAATRERAHKLAAAISAPGYEPARELTVDDLSPDETGYMAPLSAEPNAPAILLYTSGSTGTPKGVLLTQANFGNHIALKTDILGLKRGENVLHQSSLGFDMSLVQVFCALANGGCVVIVPQDARRDPVELTSLMAQHKVSLTIATPSEYLAWLQYGSDSLAQATSWRHLCMGGEPIPQLLKDELRRLERKDLVVTNCYGPTETTAAISFQSIALDSDNHELLVDNELAKYAVGKALPNYSVRIRDPAGAWLPVNHTGEIVIGGAGVAKGYLNMPEETRARFLQTPGEDGMFYRTGDKGRLLSDGTLLCFGRINGDNQVKLRGLRIELEEVEAALLQASQGLIHTAVVSRRGDVLVAHCARSHESSDTTAAGEQQATAILRRVSELLPQYSVPAAIALLPSLPTNANAKLDRK | ADN43685.1  (Heneghan et al. 2011) |
| Ace1 | 3,088–3,579 | LTYSQMIARINDIAAKLIDAKVGTGIVGVMQASTMDFICSILAVWKAGAIYTPLDPRLNSTDRLKAVVDECQPACILVDATTKPLFDSLATNAVQIDVSMVQSSKTLEASPKVAIHAKAPSAAAVFYTSGSTGVPKGITLSHASLTYNIMAATRQFGFKEGVDIMLQQSSFSFDMALAQMLTSLSNGGTLVVVPSHLRGDALGLSQLIVAENVSIVQASPTEYKSLIGVNAQHLKTSKWRVALSGGENMTQSLLEVFRSLGKPDLVLFNGYGPTEATINANTRIVPYHEPNSNPDLPLLTWPNYSISIVDLELNPVPVGVFGEVCIGGAGVGLGYFKNDELTAKAFVADKTAPAEFVAKGWKTKFRTGDLGRLSPDGGLIIEGRIDGDTQVKLRGMRIDLKNIESAILQAGAGKIIDAAVSVRRGGADESEPQYLVGHVVLDADQTPEDSQQDFLAQLIPRLRLPRHMKPSLLVPIRALPQTASHKLDRRA | G4MVZ2.1  (Song et al. 2015) |
| ScopF | 3,201–3,711 | KTALIDAGQGSKTPSSSLTYAQMADRVNTIAAAMRNSEPPVSSGSVVGVFLQPGMEWICSLLAVLRLGAIYVPLDPRTGFSRLSVIVQDCKPAAILVNNSTEQDSKGFDSVGTRINIDQIDISNHTAVSNKAEGNSVAAILYTSGSTGVPKGIVMKHETFRNNIEIMTTESYFREGLDATLQQSSYSFDMSLSQTFVTLANGGTLHVVPKELRGDPTAISSIIATQGITFTIATPAEYISWIRDGNADLGKSDWVLAQTGGEPVSKALANSFQEVGKASLKLVDCYGPTEITFCCGSRDVDYWAETSDKRHQDVDGANVAGLKTWPNYSVCIVDANTKPVPAGVPGEVLIGGAGVVAGYLHSELDARGFSRDSFASTEFLESGWSRLHRTGDLGKLSRVDGSLTLLGRIAGDTQVKLRGLRIDLKEVEAAIIDTAEGKIADAAVTVRESDTTGSEFLVAFVTTTASHGDANSDFSDVLHRLPLPPYMRPAAIVHLAKLPTNVSNKIDRNA | WWS34688 |


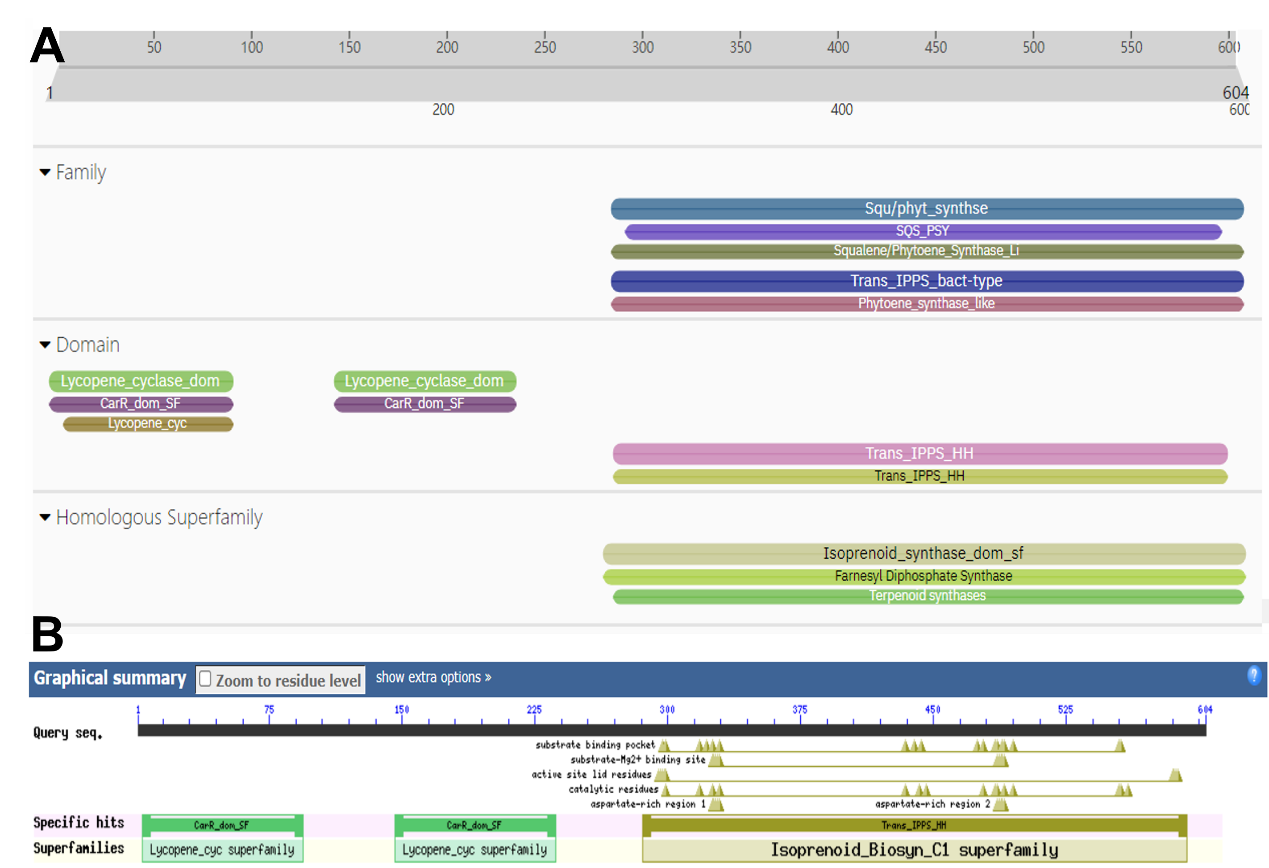


**Figure S1. Domain analyses of TS10.** The domain analysis of TS10 (GenBank accession number: PP432636) was done by Interpro (A, https://www.ebi.ac.uk/interpro) and NCBI CCD (B, https://www.ncbi.nlm.nih.gov/Structure/cdd/wrpsb.cgi).


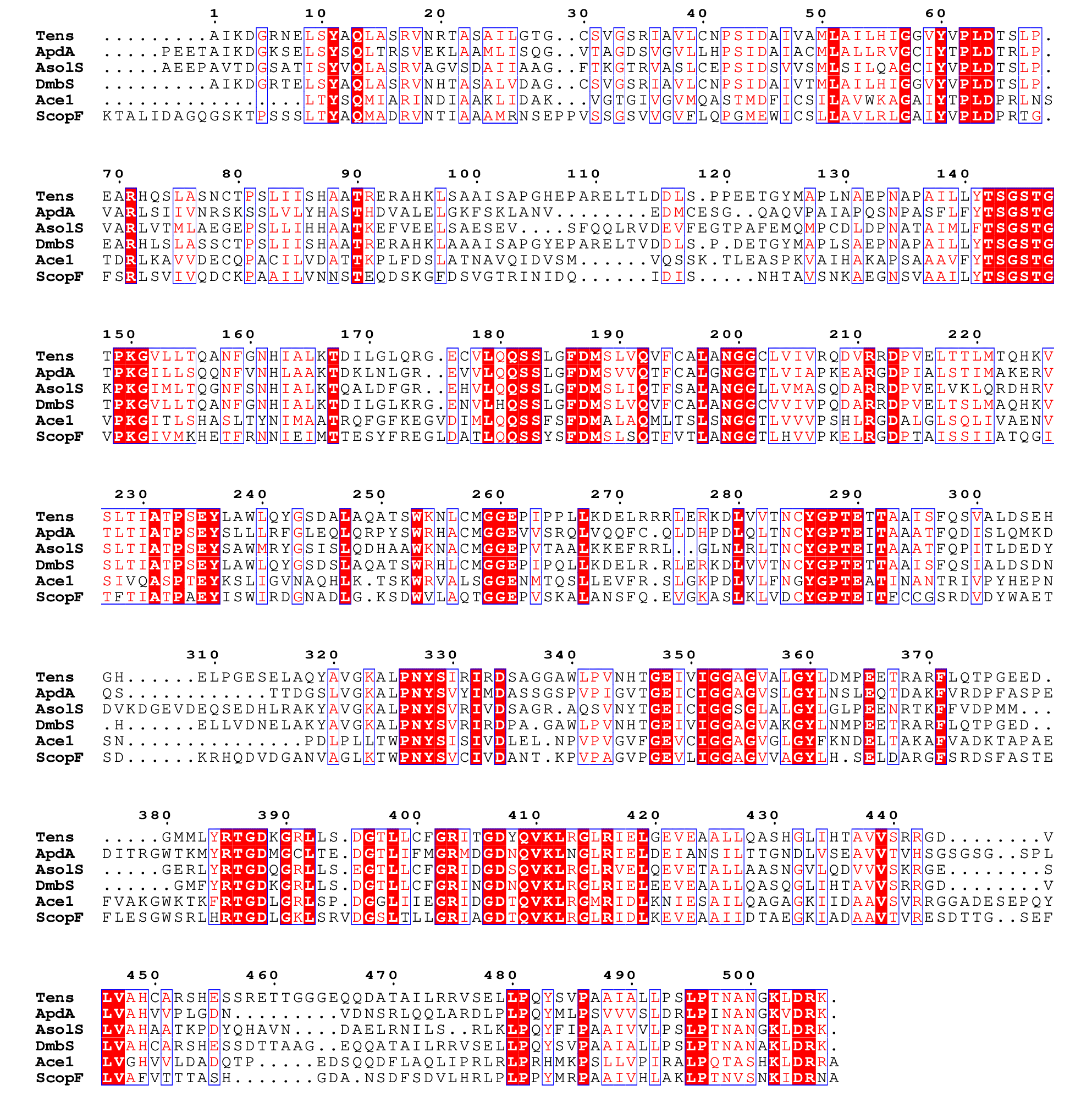


**Figure S2.** **Alignment of the tyrosine-specific A domains in selected PKS-NRPSs.**

**References**

Bergmann S, Schümann J, Scherlach K, Lange C, Brakhage AA, Hertweck C. 2007. Genomics-driven discovery of PKS-NRPS hybrid metabolites from *Aspergillus nidulans*. Nat Chem Biol. 3(4):213–217. doi:10.1038/nchembio869.

Ciavatta ML, Lopez-Gresa MP, Gavagnin M, Nicoletti R, Manzo E, Mollo E, Guo Y, Cimino G. 2008. Cytosporin-related compounds from the marine-derived fungus *Eutypella scoparia*. Tetrahedron. 64(22):5365–5369. doi:10.1016/j.tet.2008.03.016.

Defrancq E, Gordon J, Brodard A, Tabacchi R. 1992. The synthesis of a novel epoxycyclohexane from the fungus *Eutypa lata* (Pers: F.) TUL. Helv Chim Acta. 75(1):276–281. doi:10.1002/hlca.19920750123.

Eley KL, Halo LM, Song Z, Powles H, Cox RJ, Bailey AM, Lazarus CM, Simpson TJ. 2007. Biosynthesis of the 2-pyridone tenellin in the insect pathogenic fungus *Beauveria bassiana*. Chembiochem. 8(3):289–297. doi:10.1002/cbic.200600398.

Haga A, Tamoto H, Ishino M, Kimura E, Sugita T, Kinoshita K, Takahashi K, Shiro M, Koyama K. 2013. Pyridone alkaloids from a marine-derived fungus, *Stagonosporopsis cucurbitacearum*, and their activities against azole-resistant *Candida albicans*. J Nat Prod. 76(4):750–754. doi:10.1021/np300876t.

Halo LM, Marshall JW, Yakasai AA, Song Z, Butts CP, Crump MP, Heneghan M, Bailey AM, Simpson TJ, Lazarus CM, et al. 2008. Authentic heterologous expression of the tenellin iterative polyketide synthase nonribosomal peptide synthetase requires coexpression with an enoyl reductase. Chembiochem. 9(4):585–594. doi:10.1002/cbic.200700390.

Heneghan MN, Yakasai AA, Williams K, Kadir KA, Wasil Z, Bakeer W, Fisch KM, Bailey AM, Simpson TJ, Cox RJ, et al. 2011. The programming role of *trans*-acting enoyl reductases during the biosynthesis of highly reduced fungal polyketides. Chem Sci. 2(5):972–979. doi:10.1039/C1SC00023C.

Isaka M, Palasarn S, Lapanun S, Chanthaket R, Boonyuen N, Lumyong S. 2009. *γ*-lactones and *ent*-eudesmane sesquiterpenes from the endophytic fungus *Eutypella* sp. BCC 13199. J Nat Prod. 72(9):1720–1722. doi:10.1021/np900316x.

Isaka M, Palasarn S, Prathumpai W, Laksanacharoen P. 2011. Pimarane diterpenes from the endophytic fungus *Eutypella* sp. BCC 13199. Chem Pharm Bull. 59(9):1157–1159. doi:10.1248/cpb.59.1157.

Kongprapan T, Rukachaisirikul V, Saithong S, Phongpaichit S, Poonsuwan W, Sakayaroj J. 2015. Cytotoxic cytochalasins from the endophytic fungus *Eutypella scoparia* PSU-H267. Phytochem Lett. 13:171–176. doi:10.1016/j.phytol.2015.06.010.

Kuriakose GC, Palem PPC, Jayabaskaran C. 2016. Fungal vincristine from *Eutypella* spp - CrP14 isolated from *Catharanthus roseus* induces apoptosis in human squamous carcinoma cell line -A431. BMC Complement Altern Med. 16(1):302. doi:10.1186/s12906-016-1299-2.

Liao H, Sun D, Zheng C, Wang C. 2017. A new hexahydrobenzopyran derivative from the gorgonian-derived fungus *Eutypella* sp. Nat Prod Res. 31(14):1640–1646. doi:10.1080/14786419.2017.1285301.

Lin Y, Li H, Jiang G, Zhou S, Vrijmoed LLP, Jones EBG. 2002. A novel *γ*-lactone, eutypoid-A and other metabolites from marine fungus *Eutypa* sp. (#424) from the South China Sea. Indian J Chem, Sect B: Org Chem Incl Med Chem. 41B:1542–1544. doi:10.1002/chin.200245200.

Liu H, Zhang L, Chen Y, Sun Z, Pan Q, Li H, Zhang W. 2017a. Monoterpenes and sesquiterpenes from the marine sediment-derived fungus *Eutypella scoparia* FS46. J Asian Nat Prod Res. 19(2):145–151. doi:10.1080/10286020.2016.1189906.

Liu H, Zhang L, Chen Y, Li S, Tan G, Sun Z, Pan Q, Ye W, Li H, Zhang W. 2017b. Cytotoxic pimarane-type diterpenes from the marine sediment-derived fungus *Eutypella* sp. FS46. Nat Prod Res. 31(4):404–410. doi:10.1080/14786419.2016.1169418.

Liu J, Hu B, Gao Y, Zhang J, Jiao B, Lu X, Liu X. 2014. Bioactive tyrosine-derived cytochalasins from fungus *Eutypella* sp. D-1. Chem Biodivers. 11(5):800–806. doi:10.1002/cbdv.201300218.

Liu X. 2016. Researches on structure elucidation and biological activities investigation of secondary metabolites of two polar fungi [dissertation]. Shanghai (CN): Naval Medical University.

Lu X, Liu J, Liu X, Gao Y, Zhang J, Jiao B, Zheng H. 2014. Pimarane diterpenes from the Arctic fungus *Eutypella* sp. D-1. J Antibiot (Tokyo). 67(2):171–174. doi:10.1038/ja.2013.104.

Lu X, Liu X, Liu J, Chen B, Jiao B, Zhang Y, Cao M, Dong Q, inventors; Zhao, Qing, assignee. May 20, 2015. A sesquiterpene lactone with antimicrobial activity and its application. China patent CN 104628680 A.

Molyneux RJ, Mahoney N, Bayman P, Wong RY, Meyer K, Irelan N. 2002. *Eutypa* dieback in grapevines: differential production of acetylenic phenol metabolites by strains of *Eutypa lata*. J Agric Food Chem. 50(6):1393–1399. doi:10.1021/jf011215a.

Ning Y, Zhang S, Zheng T, Xu Y, Li S, Zhang J, Jiao B, Zhang Y, Ma Z, Lu X. 2023. Pimarane-type diterpenes with anti-inflammatory activity from Arctic-derived fungus *Eutypella* sp. D-1. Mar Drugs. 21(10):541. doi:10.3390/md21100541.

Niu S, Liu D, Shao Z, Liu J, Fan A, Lin W. 2021. Chemical epigenetic manipulation triggers the production of sesquiterpenes from the deep-sea derived *Eutypella* fungus. Phytochemistry. 192:112978. doi:10.1016/j.phytochem.2021.112978.

Niu S, Liu D, Shao Z, Proksch P, Lin W. 2017a. Eutypellazines A–M, thiodiketopiperazine-type alkaloids from deep sea derived fungus *Eutypella* sp. MCCC 3A00281. RSC Adv. 7(53):33580–33590. doi:10.1039/C7RA05774A.

Niu S, Liu D, Shao Z, Proksch P, Lin W. 2017b. Eutypellazines N−S, new thiodiketopiperazines from a deep sea sediment derived fungus *Eutypella* sp. with anti-VRE activities. Tetrahedron Lett. 58(38):3695–3699. doi:10.1016/j.tetlet.2017.08.015.

Niu S, Liu D, Shao Z, Proksch P, Lin W. 2018. Eremophilane-type sesquiterpenoids in a deep-sea fungus *Eutypella* sp. activated by chemical epigenetic manipulation. Tetrahedron. 74(51):7310–7325. doi:10.1016/j.tet.2018.10.056.

Oh H, Jensen PR, Murphy BT, Fiorilla C, Sullivan JF, Ramsey T, Fenical W. 2010. Cryptosphaerolide, a cytotoxic Mcl-1 inhibitor from a marine-derived ascomycete related to the genus *Cryptosphaeria*. J Nat Prod. 73(5):998–1001. doi:10.1021/np1000889.

Perez-Gonzalez G, Sebestyen D, Petit E, Jellison J, Mugnai L, Gelhaye E, Lee N, Farine S, Bertsch C, Goodell B. 2022. Oxygen radical-generating metabolites secreted by *Eutypa* and *Esca* fungal consortia: understanding the mechanisms behind grapevine wood deterioration and pathogenesis. Front Plant Sci. 13:921961. doi:10.3389/fpls.2022.921961.

Pongcharoen W, Rukachaisirikul V, Phongpaichit S, Rungjindamai N, Sakayaroj J. 2006. Pimarane diterpene and cytochalasin derivatives from the endophytic fungus *Eutypella scoparia* PSU-D44. J Nat Prod. 69(5):856–858. doi:10.1021/np0600649.

Qi S, Wang Y, Zheng Z, Xu Q, Deng X. 2015. Cytochalasans and sesquiterpenes from *Eutypella scoparia* 1-15. Nat Prod Commun. 10(12):2027–2030. doi:10.1177/1934578X1501001203.

Renaud J, Tsoupras G, Stoeckli-Evans H, Tabacchi R. 1989a. A novel allenic epoxycyclohexane and related compounds from *Eutypa lata* (Pers: F.) Tul. Helv Chim Acta. 72(6):1262–1267. doi:10.1002/hlca.19890720612.

Renaud J, Tsoupras G, Tabacchi R. 1989b. Biologically active natural acetylenic compounds from *Eutypa lata* (Pers: F.) TUL. Helv Chim Acta. 72(5):929–932. doi:10.1002/hlca.19890720508.

Song Z, Bakeer W, Marshall JW, Yakasai AA, Khalid RM, Collemare J, Skellam E, Tharreau D, Lebrun M-H, Lazarus CM et al. 2015. Heterologous expression of the avirulence gene ACE1 from the fungal rice pathogen *Magnaporthe oryzae*. Chem Sci. 6(8):4837–4845. doi:10.1039/C4SC03707C.

Sun L, Li D, Chen Y, Tao M, Dan F, Zhang W. 2011a. Secondary metabolites of marine fungus *Eutypella scoparia* from the South China Sea and their antitumor activities. Chin Tradit Herb Drugs. 42(3):432–436.

Sun L, Li D, Chen Y, Tao M, Zhang W, Dan F. 2011b. Purification,identification and antitumor activities of secondary metabolites from marine fungus *Eutypella scoparia* FS26. Mycosystema. 30(2):268–274. doi:10.13346/j.mycosystema.2011.02.021.

Sun L, Li D, Tao M, Dan F, Zhang W. 2012a. Two new sesquiterpenes from the marine fungus *Eutypella scoparia* FS26 from the South China Sea. Helv Chim Acta. 95(1):157–162. doi:10.1002/hlca.201100275

Sun L, Li D, Tao M, Chen Y, Dan F, Zhang W. 2012b. Scopararanes C–G: new oxygenated pimarane diterpenes from the marine sediment-derived fungus *Eutypella scoparia* FS26. Mar Drugs. 10(3):539–550. doi:10.3390/md10030539.

Sun L, Li D, Tao M, Chen Y, Zhang Q, Dan F, Zhang W. 2013. Two new polyketides from a marine sediment-derived fungus *Eutypella scoparia* FS26. Nat Prod Res. 27(14):1298–1304. doi:10.1080/14786419.2012.733393.

Tan J, Liu X, Zhang J, Chen X, Li Y, Jiao B. 2017. Crystal structure of (*R*)-1-(2,3-dihydro-1*H*-pyrrolizin-5-yl)-2,3-dihydroxypropan-1-one, C10H13NO3. Z KRIST-NEW CRYST ST. 232(1):7–8. doi:10.1515/ncrs-2016-0099.

Tanapichatsakul C, Pansanit A, Monggoot S, Brooks S, Prachya S, Kittakoop P, Panuwet P, Pripdeevech P. 2020. Antifungal activity of 8-methoxynaphthalen-1-ol isolated from the endophytic fungus *Diatrype palmicola* MFLUCC 17-0313 against the plant pathogenic fungus *Athelia rolfsii* on tomatoes. PeerJ. 8:e9103. doi:10.7717/peerj.9103.

Vicente F, Basilio A, Platas G, Collado J, Bills GF, González Del Val A, Martín J, Tormo JR, Harris GH, Zink DL et al. 2009. Distribution of the antifungal agents sordarins across filamentous fungi. Mycol Res. 113(6–7):754–770. doi:10.1016/j.mycres.2009.02.011.

Wang X, Sun K, Wang B. 2018. Bioactive pimarane diterpenes from the Arctic fungus *Eutypella* sp. D-1. Chem Biodiversity. 15(2):e1700501. doi:10.1002/cbdv.201700501.

Wang Y, Wang Y, Wu A, Zhang L, Hu Z, Huang H, Xu Q, Deng X. 2017. New 12,8-eudesmanolides from *Eutypella* sp. 1-15. J Antibiot (Tokyo). 70(10):1029–1032. doi:10.1038/ja.2017.89.

Williams RB, Henrikson JC, Hoover AR, Lee AE, Cichewicz RH. 2008. Epigenetic remodeling of the fungal secondary metabolome. Org Biomol Chem. 6(11):1895–1897. doi:10.1039/B804701D.

Xu W, Cai X, Jung ME, Tang Y. 2010. Analysis of intact and dissected fungal polyketide synthase-nonribosomal peptide synthetase in vitro and in *Saccharomyces cerevisiae*. J Am Chem Soc. 132(39):13604–13607. doi:10.1021/ja107084d.

Yoshida S, Kito K, Ooi T, Kanoh K, Shizuri Y, Kusumi T. 2007. Four pimarane diterpenes from marine fungus: chloroform incorporated in crystal lattice for absolute configuration analysis by X-ray. Chem Lett. 36(11):1386–1387. doi:10.1246/cl.2007.1386.

Yu H, Ning Z, Hu B, Zhu Y, Lu X, He Y, Jiao B, Liu X. 2023. Cytosporin derivatives from Arctic-derived fungus *Eutypella* sp. D-1 via the OSMAC approach. Mar Drugs. 21(7):382. doi:10.3390/md21070382.

Yu H, Wang X, Hu B, Zhang J, Lu X, Liu X, Jiao B. 2020. Studies on bioactive components of the Arctic fungus *Eutypella* sp. D-1. Chin J Mar Drugs. 39(2):38–41. doi:10.13400/j.cnki.cjmd.2020.02.005.

Yu H, Wang X, Xu W, Zhang Y, Qian Y, Zhang J, Lu X, Liu X. 2018a. Eutypellenoids A-C, new pimarane diterpenes from the Arctic fungus *Eutypella* sp. D-1. Mar Drugs. 16(8):284. doi:10.3390/md16080284.

Yu H, Wang X, Zhang Y, Xu W, Zhang J, Zhou X, Lu X, Liu X, Jiao B. 2018b. Libertellenones O-S and eutypellenones A and B, pimarane diterpene derivatives from the Arctic fungus *Eutypella* sp. D-1. J Nat Prod. 81(7):1553–1560. doi:10.1021/acs.jnatprod.8b00039.

Zhang L, Chen X, Chen Z, Wang G, Zhu S, Yang Y, Chen K, Liu X, Li Y. 2016. Eutypenoids A-C: novel pimarane diterpenoids from the Arctic fungus *Eutypella* sp. D-1. Mar Drugs. 14(3):44. doi:10.3390/md14030044.

Zhang W, Lu X, Huo L, Zhang S, Chen Y, Zou Z, Tan H. 2021a. Sesquiterpenes and steroids from an endophytic *Eutypella scoparia*. J Nat Prod. 84(6):1715–1724. doi:10.1021/acs.jnatprod.0c01167.

Zhang W, Lu X, Wang H, Chen Y, Zhang J, Zou Z, Tan H. 2021b. Antibacterial secondary metabolites from the endophytic fungus *Eutypella scoparia* SCBG-8. Tetrahedron Lett. 79:153314. doi:10.1016/j.tetlet.2021.153314.

Zhang W, Wang M, Zhang S, Xu K, Tan G, Qiu S, Zou Z, Tan H. 2020. Eutyscoparols A-G, polyketide derivatives from endophytic fungus *Eutypella scoparia* SCBG-8. Fitoterapia. 146:104681. doi:10.1016/j.fitote.2020.104681.

Zhang Y, Du H, Gao W, Li W, Cao F, Wang C. 2022. Anti-inflammatory polyketides from the marine-derived fungus Eutypella scoparia. Mar Drugs. 20(8):486. doi:10.3390/md20080486.

Zhang Y, Yu H, Xu W, Hu B, Guild A, Zhang J, Lu X, Liu X, Jiao B. 2019. Eutypellacytosporins A-D, meroterpenoids from the Arctic fungus *Eutypella* sp. D-1. J Nat Prod. 82(11):3089–3095. doi:10.1021/acs.jnatprod.9b00700.

Zhou Y, Zhang Y, Zhang J, Yu H, Liu X, Lu X, Jiao B. 2017. A new sesquiterpene lactone from fungus *Eutypella* sp. D-1. Nat Prod Res. 31(14):1676–1681. doi:10.1080/14786419.2017.1286486.
